# The anxiolytic effect of probiotics: A systematic review and meta-analysis of the clinical and preclinical literature

**DOI:** 10.1371/journal.pone.0199041

**Published:** 2018-06-20

**Authors:** Daniel J. Reis, Stephen S. Ilardi, Stephanie E. W. Punt

**Affiliations:** Department of Psychology, University of Kansas, Lawrence, KS, United States of America; McMaster University, CANADA

## Abstract

**Background:**

Probiotics have generated intensive research interest in recent years as a novel mode of treatment for physical and mental illness. Nevertheless, the anxiolytic potential of probiotics remains unclear. The present systematic review and meta-analysis aimed to evaluate the clinical and preclinical (animal model) evidence regarding the effect of probiotic administration on anxiety.

**Methods:**

The PubMed, PsycINFO, and Web of Science databases were reviewed for preclinical and clinical studies that met the defined inclusion and exclusion criteria. The effects of probiotics on anxiety-like behavior and symptoms of anxiety were analyzed by meta-analyses. Separate subgroup analyses were conducted on diseased versus healthy animals, specific preclinical probiotic species, and clinical versus healthy human samples.

**Results:**

Data were extracted from 22 preclinical studies (743 animals) and 14 clinical studies (1527 individuals). Overall, probiotics reduced anxiety-like behavior in animals (Hedges’ g = -0.47, 95% CI -0.77 –-0.16, *p* = 0.004). Subgroup analyses revealed a significant reduction only among diseased animals. Probiotic species-level analyses identified only *Lactobacillus* (*L*.) *rhamnosus* as an anxiolytic species, but these analyses were broadly under-powered. Probiotics did not significantly reduce symptoms of anxiety in humans (Hedges’ g = -0.12, 95% CI -0.29–0.05, *p* = 0.151), and did not differentially affect clinical and healthy human samples.

**Conclusions:**

While preclinical (animal) studies suggest that probiotics may help reduce anxiety, such findings have not yet translated to clinical research in humans, perhaps due to the dearth of extant research with clinically anxious populations. Further investigation of probiotic treatment for clinically relevant anxiety is warranted, particularly with respect to the probiotic species *L*. *rhamnosus*.

## Introduction

Anxiety disorders are a class of psychological disturbances characterized by pervasive worry, fear, and related behavioral impairments. Collectively, they are the most prevalent form of mental illness [1]—affecting up to 30% of American adults at some point—and they impose a large societal burden of functional disability and mortality [2]. Excessive anxiety is also associated with numerous negative health outcomes, such as increased risk of coronary heart disease [3], impaired sleep [4], and alcohol and substance abuse [5]. Although there now exist several established medication- and psychotherapy-based treatments for anxiety [6], many patients still experience a poor treatment response [7, 8]. The widespread and debilitating nature of anxiety, in tandem with the frequent inadequacy of existing treatments, points to the desirability of exploring and developing novel approaches to treatment.

One particularly promising area of investigation involves manipulation of the intestinal microbiota, the diverse collection of symbiotic microorganisms residing within the human gut [9]. The microbiota communicates with the central nervous system via a collection of bidirectional neural, metabolic, and immune pathways known as the microbiota-gut-brain axis [10]. Microbiota dysfunction—most commonly, the relative loss of beneficial gut microbes—is associated with numerous types of physical and mental illness, ranging from irritable bowel syndrome to Alzheimer’s disease to depression [11]. The experience of anxiety is closely interrelated with disordered gut function, to such an extent that commonly reported symptoms of anxiety often involve intestinal distress (e.g. upset stomach or nausea), and the severity and duration of abdominal pain are associated with elevated anxiety [12]. Moreover, anxiety frequently co-occurs with gastrointestinal disorders, such as irritable bowel syndrome, Crohn’s disease, and ulcerative colitis [13, 14]—all of which are also linked with microbiota dysfunction [11]. Antibiotic use, which can profoundly reduce the gut’s bacterial diversity [15], has also been found to increase the risk of developing an anxiety disorder later in life [16]. Finally, gastrointestinal disturbance caused by pathogens can elicit anxiety. Intestinal infections in humans are associated with increased risk of developing an anxiety disorder over the next two years [17], and healthy mice infected with a foodborne pathogen have been shown to rapidly display increased anxiety-like behavior [18], even in the absence of a detectable immune response [19], suggesting that such microorganisms can directly interact with neural pathways.

The most common way of addressing microbiota dysfunction and associated illness is through the supplemental administration of *probiotics* (beneficial microorganisms). Recent meta-analyses have found that probiotic intervention successfully reduces symptoms of both irritable bowel syndrome [20] and ulcerative colitis [21]. Probiotics are even emerging as a recommended treatment for antibiotic-associated adverse events in children [22]. Additionally, there is early evidence that probiotics may have psychotropic effects. Tillisch et al. [23], for example, demonstrated that the consumption of probiotics altered emotional processing in the brains of healthy women. Probiotics have also been shown to improve self-reported mood in otherwise healthy adults experiencing negative affect [24]. And animal studies have found that pretreatment with probiotics can protect against the neurological damage induced by both acute and chronic stress [25, 26]. Given that these findings came from healthy humans (and animals), they suggest that probiotics may have beneficial effects even in the absence of clinical disease. This leaves open the possibility that probiotics may be useful for both disease prevention and treatment via different underlying mechanisms.

Several recent reviews have summarized the extant literature regarding probiotics and anxiety [27–29]. Probiotics appear to be capable of reducing anxiety-like behavior in animals [29], although the impact of probiotics on anxiety in humans is less certain, with recent narrative reviews arriving at differing conclusions [27–29]. Notably, the overall effect of probiotics on anxiety has yet to be *quantified* for either preclinical or clinical research. Accordingly, the goal of this study was to comprehensively summarize and quantify the existing evidence on the relationship between probiotics and anxiety. To do so, systematic reviews and meta-analyses were performed on both preclinical and clinical studies, respectively.

## Methods

The preclinical and clinical reviews followed CAMARADES and PRISMA guidelines for conducting systematic reviews and meta-analyses, respectively [30, 31]. The study was not preregistered, and the protocol can be viewed at https://dx.doi.org/10.17504/protocols.io.nsadeae. Inclusion and exclusion criteria were selected to maximize the acquisition of all possible studies that examined the effects of probiotic administration on anxiety-like behavior in rodents or symptoms of anxiety in humans. Conference abstracts were omitted due to a lack of necessary information.

### Preclinical criteria

Preclinical studies were deemed eligible if they met the following inclusion criteria: 1) subjects were either rats or mice; 2) a probiotic was experimentally administered; 3) anxiety-like behavior was measured.

Studies that met one or more of the following criteria were excluded: 1) there was no matched control group; 2) the probiotic was not living at time of administration (e.g. heat-killed); 3) the probiotic was not administered directly to the tested subject (e.g. administered to the mother of infant rodent); 4) means, standard deviations, and sample sizes were not available for the measured anxiety-like behavior; 5) the full text of the study was not available in English.

### Clinical criteria

Clinical studies were deemed eligible if they met the following inclusion criteria: 1) the study described a randomized controlled trial; 2) at least one interventional arm administered a probiotic; 3) an anxiety scale was used as a primary or secondary measure; 4) human participants were included.

Studies that met one or more of the following criteria were excluded: 1) there was no matched control group; 2) the probiotic was not living at time of administration (e.g. heat-killed); 3) means, standard deviations, and sample sizes were not available for the anxiety measurements; 4) the full text of the study was not available in English.

### Search strategy

**Fig 1:**
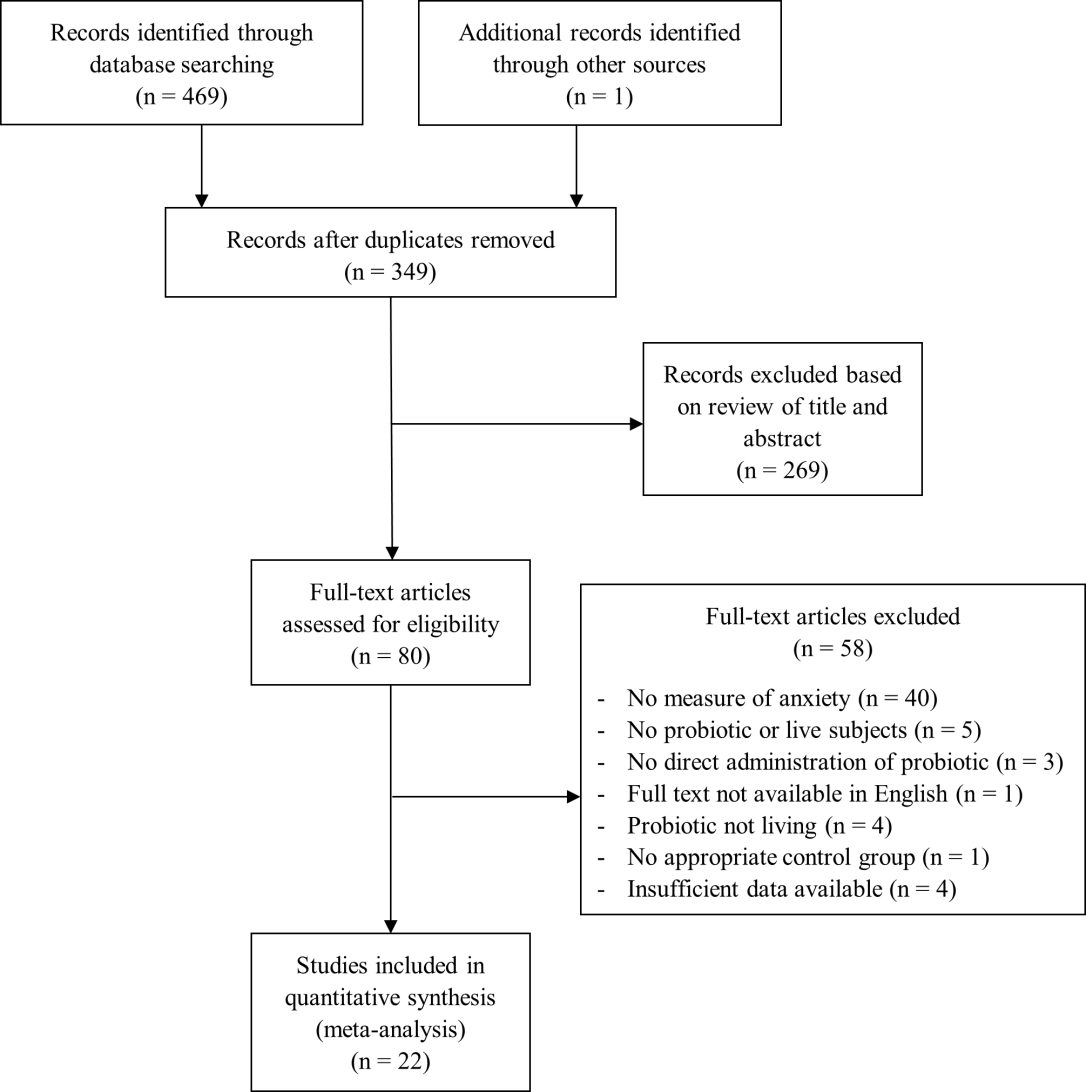
Flow and selection of preclinical studies.

**Fig 2:**
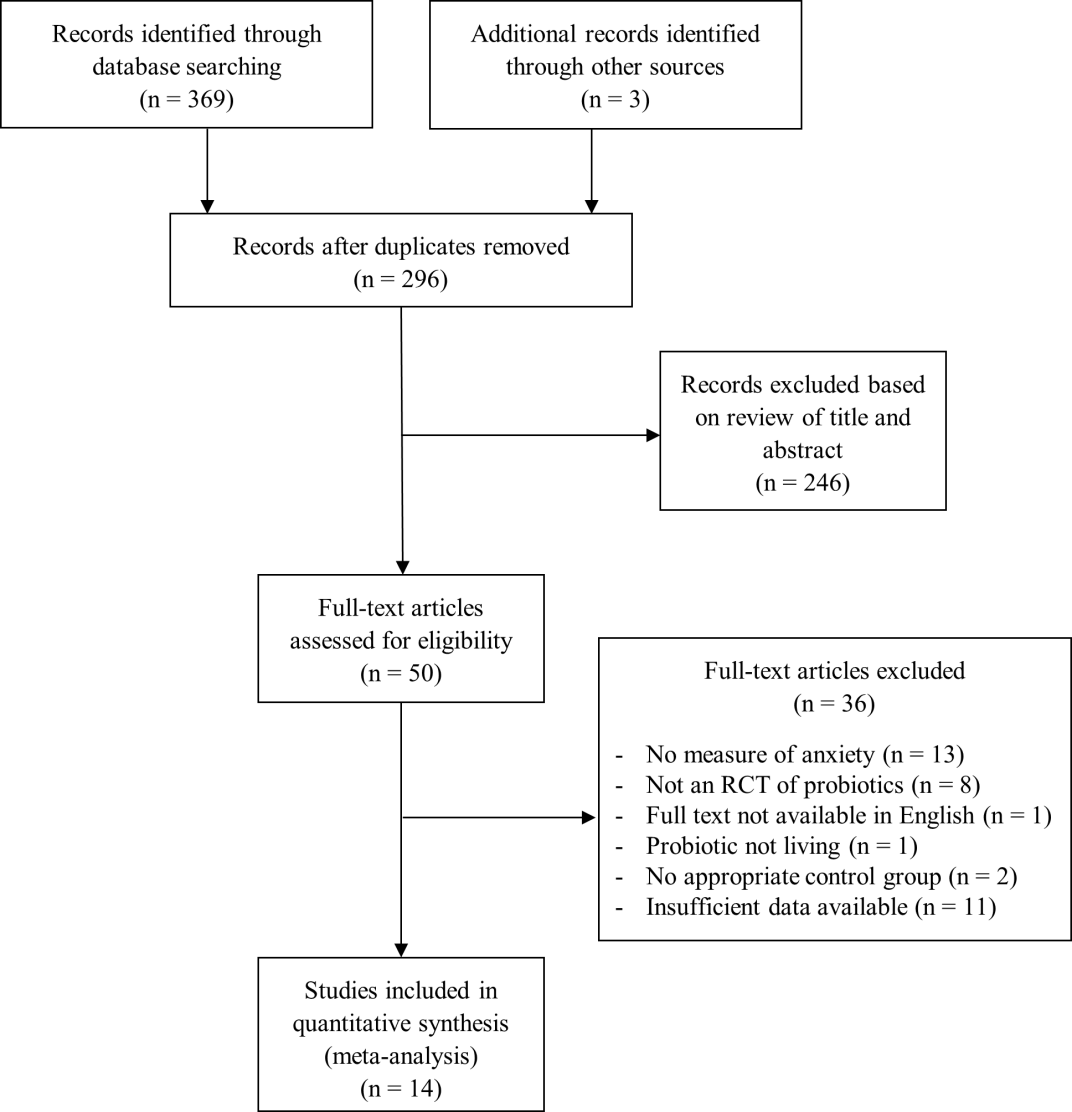
Flow and selection of clinical studies.

The systematic literature reviews were carried out using PubMed, PsycINFO, and Web of Science databases, from the earliest record of the databases to November 2017. Search terms included Bifidobacterium OR lactobacillus OR probiotic AND anxiety (see S1 Appendix for the exemplar PubMed preclinical and clinical search strategies). Relevant references from the identified publications were also included. The title and abstract for each search result were then evaluated to identify potential studies, and, finally, full-texts were evaluated to determine study inclusion. Screening and evaluation were performed in a standardized manner by two independent reviewers (DR and SP). Disagreements during this process were resolved according to the following process: 1) both reviewers independently reapplied the inclusion/exclusion criteria to the study in question; 2) the two reviewers discussed the criteria until a consensus was reached; 3) in cases for which a consensus could not be reached, it was planned for the final decision to be made by an independent party (SI), although this was not necessary. The flow charts of study selection can be viewed in Figs 1 and 2.

### Data collection

Preclinical and clinical data was extracted from selected studies using custom forms and included the following information based on CAMARADES and PRISMA guidelines [30, 31]: 1) study design characteristics, such as subject information (e.g. age, sex, health status, rodent species) and type of intervention (e.g. probiotic composition, dosage, and duration); and 2) outcome data (e.g. outcome measure, group sample sizes, mean value of effect, and group variance). Outcome data was included if it was derived from a measure of anxiety-like behavior or anxiety symptoms—equivalency across measures was assumed for each meta-analysis. Data for the final measurement of the interventional period was selected if the outcome was measured at multiple time periods. Potential study bias was evaluated using SYRCLE’s risk of bias tool [32] for preclinical studies and the Cochrane Collaboration’s risk of bias tool [33] for clinical studies. One reviewer (DR) extracted all included data, which was checked and confirmed by a second reviewer (SP). Disagreements between reviewers during data extraction were resolved by discussion.

One clinical study [34] reported only median values and interquartile range. In order to calculate an SMD, the median value of the reported data was assumed to represent the mean and the standard deviation was calculated by dividing the interquartile range by 1.35 [35].

When results were available only in graphical format, data was extracted using WebPlotDigitizer graph digitization software [36]; graph digitization has been previously shown to be a valid method for extracting study data [37], and WebPlotDigitizer has been recommend for use in systematic reviews [38].

Finally, nine preclinical and 14 clinical authors were contacted and asked to provide further information. Four preclinical and two clinical authors responded and provided additional study data, which were included in the final study selection. Extracted data can be viewed in Tables 1 and 2.

**Table 1:** Preclinical study characteristics.

Author, year	Subjects	No. of subjects	Days of intervention	Bacterial species and dosage (CFU)	Anxiety measure
Agusti A, 2017 Group 1	Male C57BL-6 mice	20	98	*B*. *pseudocatenulatum* CECT 7765 (1 x 10^9^)	Open field test: ↔ time in center; Light-dark test: ↔ latency
Agusti A, 2017 Group 2	Male C57BL-6 mice fed a high-fat diet	20	98	*B*. *pseudocatenulatum* CECT 7765 (1 x 10^9^)	Open field test: ↔ time in center; Light-dark test: ↔ latency
Barrera-Bugueno C, 2017	Male Sprague-Dawley rats	18	14	*L*. *casei* 54-2-33 (1 x 10^4^ CFU/mL of drinking water)	Open field test: ↑ time in center, ↑ entries into center; Elevated plus maze: ↔ time in open arms, ↔ entries into open arms
Beilharz J, 2017 Group 1	Male Sprague-Dawley rats	30	36	*B*. *longum* DSM 24736, *B*. *infantis* DSM 24737, *B*. *breve* DSM 24732, *L*. *acidophilus* DSM 24735, *L*. *paracasei* DSM 24733, *L*. *bulgaricus* DSM 24734, *L*. *plantarum* DSM 24730, *Streptococcus thermophilus subsp*. *thermophilus* DSM 24731 (Low dose– 2.5 x 10^9^; High dose– 2.5 x 10^10^)	Elevated plus maze: ↔ time in open arms
Beilharz J, 2017 Group 2	Male Sprague-Dawley rats fed a cafeteria diet	29	36	*B*. *longum* DSM 24736, *B*. *infantis* DSM 24737, *B*. *breve* DSM 24732, *L*. *acidophilus* DSM 24735, *L*. *paracasei* DSM 24733, *L*. *bulgaricus* DSM 24734, *L*. *plantarum* DSM 24730, *Streptococcus thermophilus subsp*. *thermophilus* DSM 24731 (Low dose– 2.5 x 10^9^; High dose– 2.5 x 10^10^)	Elevated plus maze: ↔ time in open arms
Bercik P, 2010	Male AKR mice infected with *Trichuris muris*	42	30	*L*. *rhamnosus* NCC4007 *and B*. *longum* NCC300*1* (1 x 10^10^)	Light-dark box test: ↓ time in light box, ↔ latency to re-enter light box; Step-down test: ↓ latency
Bercik P, 2011 Group 1	Male AKR mice exposed to dextran sodium sulfate	23	14	*B*. *longum* (1 x 10^10^)	Step-down test: ↓ latency
Bercik P, 2011 Group 2	Vagotomized male AKR mice exposed to dextran sodium sulfate	30	14	*B*. *longum* (1 x 10^10^)	Step-down test: ↔ latency
Bharwani A, 2017 Group 1	Male C57BL/6 mice	30	28	*L*. *rhamnosus* JB-1 (1.67 x 10^9^)	Light-dark box test: ↔ entries into light zone
Bharwani A, 2017 Group 2	Male C57BL/6 mice exposed to social defeat	31	28	*L*. *rhamnosus* JB-1 (1.67 x 10^9^)	Light-dark box test: ↓ entries into light zone
Bravo J, 2011	Male BALB/c mice	36	28	*L*. *rhamnosus* JB-1 (1 x 10^9^)	Elevated plus maze: ↓ open arm entries, ↔ time in open arms
Cowan C, 2016	Female Sprague-Dawley rats	16	13	*L*. *rhamnosus* R0011 and *L*. *helveticus* R0052 (1 x 10^9^ CFU/mL of drinking water)	Elevated plus maze: ↔ open arm entries, ↔ time in open arms, ↔ latency
Divyashri G, 2015	Male CFT-Swiss mice	24	28	*Enterococcus faecium* CFR 3003 (Low dose– 1 x 10^4^; High dose– 1 x 10^8^) or *L*. *rhamnosus* GG MTCC 1408 (1 x 10^8^)	Elevated plus maze: ↓ open arm entries, ↓ time in open arms; Open field test: ↔ entries into center, ↔ time in center
Emge J, 2016 Group 1	Male and female C57BL/6 mice	10	15	*L*. *rhamnosus* R0011 and *L*. *helveticus* R0052 (2 x 10^9^)	Light-dark box test: ↔ time in light box
Emge J, 2016 Group 2	Male and female C57BL/6 mice exposed to dextran sodium sulfate	20	15	*L*. *rhamnosus* R0011 and *L*. *helveticus* R0052 (2 x 10^9^)	Light-dark box test: ↓ time in light box
Jang H, 2017	Male ICR mice exposed to immobilization stress	24	3	*B*. *adolescentis* IM38 (Low dose– 2 x 10^8^; Medium dose– 1 x 10^9^; High dose– 5 x 10^9^ CFU)	Elevated plus maze: ↓ open arm entries, ↓ time in open arms
Liang S, 2015	Male specific-pathogen-free Sprague-Dawley rats exposed to chronic restraint stress	16	22	*L*. *helveticus* NS8 (1 x 10^9^ CFU/mL of drinking water)	Elevated plus maze: ↔ open arm entries, ↓ time in open arms; Open field test: ↔ time in center
Liu W, 2016 Group 1	Male GF C57BL/BJNarl mice	20	16	*L*. *plantarum* PS128 (1 x 10^9^)	Elevated plus maze: ↓ time in open arms / time in closed arms ratio; Open field test: ↔ time in center
Liu W, 2016 Group 2	Male C57BL/6J mice	12	16	*L*. *plantarum* PS128 (1 x 10^9^)	Elevated plus maze: ↓ time in open arms; Open field test: ↓ time in center
Liu Y, 2016 Group 1	Male C57BL/6J mice exposed to early-life stress	20	28	*L*. *plantarum* PS128 (1 x 10^9^)	Elevated plus maze: ↔ time in open arms; Open field test: ↔ time in center
Liu Y, 2016 Group 2	Male C57BL/6J mice	18	28	*L*. *plantarum* PS128 (1 x 10^9^)	Elevated plus maze: ↓ time in open arms; Open field test: ↓ time in center
Luo J, 2014	Male specific-pathogen-free Sprague-Dawley rats with induced hyperammonemia	12	14	*L*. *helveticus* NS8 (1 x 10^9^)	Elevated plus maze: ↓ open arm entries, ↔ time in open arms
Mackos A, 2013 Group 1	Male outbred CD-1 mice	36	12	*L*. *reuteri* 23272 (1.5 x 10^8^)	Open field test: ↔ time spent in center
Mackos A, 2013 Group 2	Male outbred CD-1 mice exposed to prolonged-restraint	18	12	*L*. *reuteri* 23272 (1.5 x 10^8^)	Open field test: ↔ time spent in center
Matthews D, 2013	Male specific-pathogen-free BALB/c mice	18	Administered several times over 7 weeks	*Mycobacterium vaccae* 15,483 (4.5 x 10^5^)	Anxiety-like behaviors during maze task: ↔ immobilization, ↔ grooming, ↔ latency to start
McKernan D, 2010 Group 1	Male Sprague-Dawley rats	40	14	*L*. *salivarius* UCC118 (1 x 10^9^); or *B*. *infantis* 35624 (1 x 10^9^); or *B*. *breve* UCC2003 (1 x 10^9^)	Open field test: ↔ time in center
McKernan D, 2010 Group 2	Male Wistar-Kyoto rats	40	14	*L*. *salivarius* UCC118 (1 x 10^9^); or *B*. *infantis* 35624 (1 x 10^9^); or *B*. *breve* UCC2003 (1 x 10^9^)	Open field test: ↔ time in center
Moya-Perez A, 2017 Group 1	Male C57Bl/6J mice	18	20	*B*. *pseudocatenulatum* CECT 7765 (1 x 10^8^ CFU)	Elevated plus maze: ↔ time in open arms
Moya-Perez A, 2017 Group 2	Male C57Bl/6J mice exposed to early-life stress	18	20	*B*. *pseudocatenulatum* CECT 7765 (1 x 10^8^ CFU)	Elevated plus maze: ↓ time in open arms; Open field test: ↔ entries into center
Smith C, 2014 Group 1	Male and female wild-type *Rag1*^*-/-*^ mice	12	28	*L*. *rhamnosus* R0011 and *L*. *helveticus* R0052 (6 x 10^9^)	Light-dark box test: ↓ time in light box
Smith C, 2014 Group 2	Male and female wild-type *Rag1*^*-/-*^ mice exposed to water avoidance stress	10	28	*L*. *rhamnosus* R0011 and *L*. *helveticus* R0052 (6 x 10^9^)	Light-dark box test: ↓ time in light box
Vanhaecke T, 2017	Sprague-Dawley rats	12	15	*L*. *fermentum* CECT 5716 (1 x 10^9^ CFU/100g body weight)	Elevated plus maze: ↔ open arm entries
Wang T, 2015	Male Sprague-Dawley rats exposed to an antibiotic	20	30	*L*. *fermentum* NS9 (1 x 10^9^ CFU/mL of drinking water)	Elevated plus maze: ↓ open arm entries

↓ and ↑ represent a statistically significant decrease or increase (respectively) in anxiety-like behavior in at least one probiotic treatment group, while ↔ represents a nonsignificant or unclear change.

**Table 2:** Clinical study characteristics.

Author, year	Subjects (age)	No. of subjects	Intervention (days)	Bacterial species and dosage (CFU)	Anxiety scale
Kato-Kataoka A, 2016	Healthy 4^th^-grade medical students (average age ~23)	47	Milk (56)	*L*. *casei Shirota* YIT 9029 (1 x 10^11^)	↔ STAI-state
Kelly J, 2017	Healthy adults (average age 24.6, 20–33 range)	29 (crossover design)	Capsule (28)	*L*. *rhamnosus* JB-1 (1 x 10^9^)	↔ STAI-state, ↔ STAI-trait, and ↔ BAI
Lorenzo-Zuniga V, 2014	Adults with IBS with diarrhea (20–70 range)	71	Capsule (42)	*L*. *plantarum* CECT 7484, *L*. *plantarum* CECT 7485, and *Pediococcus acidilactici* CECT 7483 (Low dose– 3–6 x 10^9^; High dose– 1–3 x 10^10^)	↓ Visceral Sensitivity Index
Lyra A, 2016	Adults with IBS (18–65 range)	332	Capsule (84)	*L*. *acidophilus* NCFM (Low dose– 1 x 10^9^; High dose– 1 x 10^10^)	↔ HADS-anxiety subscale
Marcos A, 2004	Healthy students (18–23 range)	136	Milk (42)	*L*. *delbrueckii* subsp. *bulgaricus* (2 x 10^9^), *Streptococcus salivarius* subsp. *thermophilus* (2 x 10^10^), *L*. *casei* DN114001 (2 x 10^10^)	↔ STAI-state and ↔ STAI-trait
Messaoudi M, 2011	Healthy adults (average age ~43)	55	Powder (30)	*L*. *helveticus* R0052 *and B*. *longum* R0175 (3 x 10^9^)	↔ HADS-anxiety and ↔ HSCL-90 anxiety
Pinto-Sanchez M, 2017	Adults with IBS (median age ~43)	38	Powder (42)	*B*. *longum* NCC3001 (1 x 10^10^)	↔ HADS-anxiety, ↔ STAI-state, and ↔ STAI-trait
Romijn A, 2017	Adults (age 16+) with at least moderate low mood (average age ~35)	79	Powder (56)	*L*. *helveticus* R0052 *and B*. *longum* R0175 (2 x 10^10^)	↔ DASS-42 anxiety subscale
Simren M, 2010	Adults with IBS (average age ~43)	67	Yogurt (56)	*L*. *paracasei* subsp. *paracasei* F19, *L*. *acidophilus* La5, and *B*. *lactis* Bb12 (2 x 10^10^)	↔ HADS-anxiety subscale
Slykerman R, 2017	Pregnant women (average age ~34)	379	Capsule (Up to ~1 year)	*L*. *rhamnosus* HN001 (6 x 10^9^)	↓ STAI-6 item version
Steenbergen L, 2015	Healthy adults (average age ~20)	40	Powder (28)	*B*. *bifidum* W23, *B*. *lactis* W52, *L*. *acidophilus* W37, *L*. *brevis* W63, *L*. *casei* W56, *L*. *salivarius* W24, *Lactococcus lactis* W19 and W58 (5 x 10^9^)	↔ BAI
Takada M, 2016	Healthy 4^th^-grade medical students (average age ~23)	140	Milk (56)	*L*. *casei Shirota* YIT 9029 (1 x 10^11^)	↔ STAI-state
Takada M, 2017	Healthy 4^th^-grade medical students (average age ~23)	94	Milk (77)	*L*. *casei Shirota* YIT 9029 (1 x 10^11^)	↔ STAI-state
Yang H, 2016	Patients with cancer (average age ~58)	20	Capsule (14)	*Clostridium butyricum* (CFU not reported– 420 mg per capsule)	↓ HAMA

STAI = State-Trait Anxiety Inventory; BAI = Beck Anxiety Inventory; HADS = Hospital Anxiety and Depression Scale; HSCL-90 = Hopkins Symptom Checklist-90; DASS = Depression Anxiety Stress Scales; HAMA = Hamilton Anxiety Rating Scale.

↓ and ↑ represent a statistically significant decrease or increase (respectively) in anxiety-like behavior in at least one probiotic treatment group, while ↔ represents a nonsignificant or unclear change.

### Statistical analyses

The preclinical and clinical meta-analyses were performed with R 3.2.5 software [39]. All analyses were pre-specified unless otherwise stated. For each included study, the standardized mean difference (SMD; also known as Hedges’ g) between the probiotic and matched control groups was calculated for all continuous measures of anxiety-like behavior or anxiety symptoms. Confidence intervals were calculated for each SMD using a standard normal distribution. For both preclinical and clinical studies, sample size, probiotic duration, and probiotic dose were assessed as moderating variables in individual meta-regressions. Separate subgroup analyses were conducted on diseased (receiving experimental manipulations in addition to probiotic or vehicle intervention) and naïve animals (receiving only probiotic or vehicle intervention), as well as mouse and rat samples. Exploratory subgroup analyses were also performed on studies that used matching individual or combined probiotic species, provided that the probiotic was tested in at least three experimental groups. In humans, subgroup analyses were conducted on clinical (individuals with a medical or psychological illness) and healthy samples. Multiple subgroups within a single study (e.g. different rodent strains or experimental conditions) were included as independent SMDs, provided that each treatment group had a separate, matched control group. When multiple probiotic treatment groups were compared against the same control group, the results from the different probiotic groups were combined, and the SMD was calculated from the combined results [35].

If multiple measures of anxiety-like behavior or anxiety symptoms were reported in a single study, a separate SMD was calculated for each outcome. To account for the dependency between SMDs measured in the same sample, robust variance estimation (RVE) meta-analyses were used to estimate preclinical and clinical summary SMDs. RVE meta-analysis is a form of random-effects meta-analysis that has been shown to address SMD dependency when the covariances between outcomes measured in the same study are unknown [40]. In other words, RVE allows for multiple outcomes from a single study to be included in a meta-analysis as separate SMDs; the weights are adjusted accordingly (i.e. the SMDs share a single study weight). Precision (i.e. inverse variance) was used to weight SMDs.

One clinical study included in the final analysis [41] utilized a cross-over experimental design, but did not report the correlation between the interventional periods. A correlation of 0.5 was imputed to calculate the standard error of the SMD for the study. A sensitivity analysis, using alternative correlational values to calculate the standard error, revealed that the choice of correlational value did not impact the overall results of the clinical meta-analysis.

I^2^ was used to evaluate between-study heterogeneity. Values of I^2^ more than 25%, 50%, and 75% were selected to reflect low, moderate, and high heterogeneity, respectively, in accordance with guidelines described by Higgins et al. [42]. Potential publication bias was assessed via funnel plot and Egger test [43].

## Results

### Preclinical meta-analysis

#### Study selection and characteristics

Twenty-two studies [44–65] with 33 independent experimental groups and 743 rodent subjects were included in the preclinical meta-analysis (Fig 1 and Table 1). Eight studies used rats as experimental subjects, while the other 14 used mice. All but four studies [51, 53, 63, 64] reported using only male rodents. Fifteen of the 33 experimental groups modeled a form of disease and were exposed to additional manipulation, such as social defeat [49], early-life stress [57], or induced chronic colitis [47]. Thirteen studies assessed anxiety-like behavior using an elevated plus maze, five studies used a light-dark box test, two studies used a step-down test, nine studies used an open field test, and one study observed behaviors related to anxiety during a maze task (eight studies employed multiple paradigms). Species from the *Lactobacillus*, *Bifidobacterium*, *Mycobacterium*, and *Streptococcus* genera were used as probiotics.

#### Bias assessment

Table 3 shows the assessment of the risk of bias for the included studies. No study provided sufficient detail regarding performance bias or detection bias, and only one study provided detail regarding selection bias. This lack of reporting makes it difficult, if not impossible, to accurately determine risk of bias. More detail was provided regarding the risk of attrition bias, reporting bias, and other bias. Four studies had a high risk of reporting bias [49, 57, 60, 62], and three studies had a high risk of other bias [45, 53, 59]. A separate sensitivity analysis revealed that removal of these studies did not substantively impact the results. Overall, the risk of bias for each included study is unclear.

**Table 3:** Preclinical risk of bias assessment.

Study	Baseline characteristics	Incomplete outcome data	Selective reporting	Other bias
Agusti et al. 2017	Unclear	Low	Low	Low
Barrera-Bugueno et al. 2017	Unclear	Low	Low	High
Beilharz et al. 2017	Unclear	Low	Low	Low
Bercik et al. 2010	Unclear	Low	Low	Unclear
Bercik et al. 2011	Unclear	Unclear	Low	Unclear
Bharwani et al. 2017	Unclear	Unclear	High	Unclear
Bravo et al. 2011	Unclear	Unclear	Low	Low
Cowan et al. 2016	Low	Unclear	Low	Low
Divyashri et al. 2015	Unclear	Unclear	Low	Unclear
Emge et al. 2016	Unclear	Unclear	Low	High
Jang et al. 2017	Unclear	Low	Low	Low
Liang et al. 2015	Unclear	Low	Low	Low
Liu, W et al. 2016	Unclear	Low	Low	Low
Liu, Y et al. 2016	Unclear	Unclear	High	Low
Luo et al. 2014	Unclear	Low	Low	Low
Mackos et al. 2013	Unclear	Low	Low	High
Matthews et al. 2013	Unclear	Low	High	Low
McKernan et al. 2010	Unclear	Low	Low	Unclear
Moya-Perez et al. 2017	Unclear	Low	High	Low
Smith et al. 2014	Unclear	Unclear	Low	Unclear
Vanhaecke et al. 2017	Unclear	Unclear	Low	Unclear
Wang et al. 2015	Unclear	Low	Low	Unclear

Risk of bias relating to Sequence generation, Allocation concealment, Random housing, Blinding (intervention), Random outcome assessment, and Blinding (assessment) was Unclear for all included studies, and as such these domains have been omitted from the table

#### Probiotic efficacy

**Fig 3:**
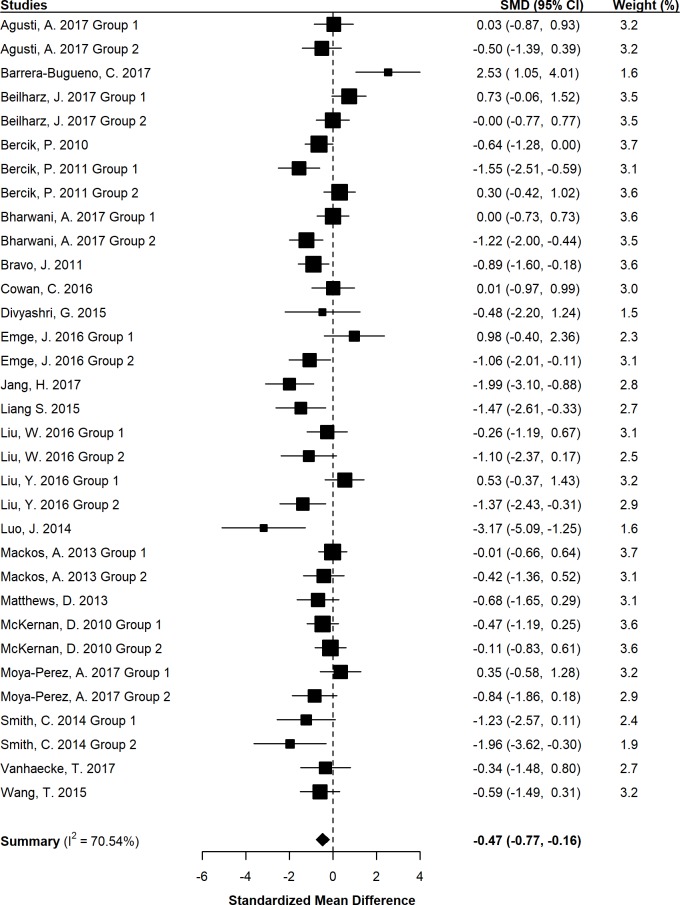
Forest plot of preclinical studies investigating the effect of probiotics on anxiety-like behavior. SMD = Standardized mean difference; CI = Confidence interval. An aggregate SMD is displayed for each experimental group. Measure-specific SMDs can be viewed in S1 Fig.

Combining standardized mean differences (SMDs) for the 33 included experimental groups revealed a pooled SMD of -0.47 (95% CI -0.77 –-0.16, *p* = 0.004; Fig 3). Probiotic administration, compared to placebo, was shown to significantly reduce anxiety-like behavior in rodents. Neither sample size (β = 0.01, 95% CI: -0.02–0.04, *p* = 0.432), probiotic duration (β = 0.01, 95% CI: -0.02–0.04, *p* = 0.372), nor probiotic dose (β = -0.006, 95% CI: -0.12–0.11, *p* = 0.906) provided a significant moderating influence.

Subgroup analyses revealed that probiotic administration significantly reduced anxiety-like behavior in diseased (SMD = -0.81, 95% CI: -1.27 - -0.35, p = 0.002), but not in naïve animals (SMD = -0.16, 95% CI: -0.58–0.26, *p* = 0.433). Furthermore, probiotics significantly reduced anxiety-like behavior in mice (SMD = -0.58, 95% CI: -0.90 –-0.26, *p* = 0.001), but not rats (SMD = -0.17, 95% CI: -1.07–0.73, *p* = 0.678).

Four probiotics were selected for additional subgroup analyses based on their utilization in multiple trials: *Lactobacillus* (*L*.) *rhamnosus*, *Bifidobacterium* (*B*.) *pseudocatenulatum*, *L*. *plantarum* (four experimental groups each), and combined *L*. *rhamnosus* and *L*. *helveticus* (five experimental groups). Only *L*. *rhamnosus* was shown to significantly reduce anxiety-like behavior (SMD = -0.77, 95% CI: -1.40 –-0.13, *p* = 0.018). Anxiety-like behavior was not affected by *B*. *pseudocatenulatum* (SMD = -0.24, 95% CI: -0.76–0.28, *p* = 0.368), *L*. *plantarum* (SMD = -0.50, 95% CI: -1.37–0.38, *p* = 0.264), or combined *L*. *rhamnosus* and *L*. *helveticus* (SMD = -0.61, 95% CI: -1.54–0.32, *p* = 0.201).

#### Publication bias and heterogeneity

**Fig 4:**
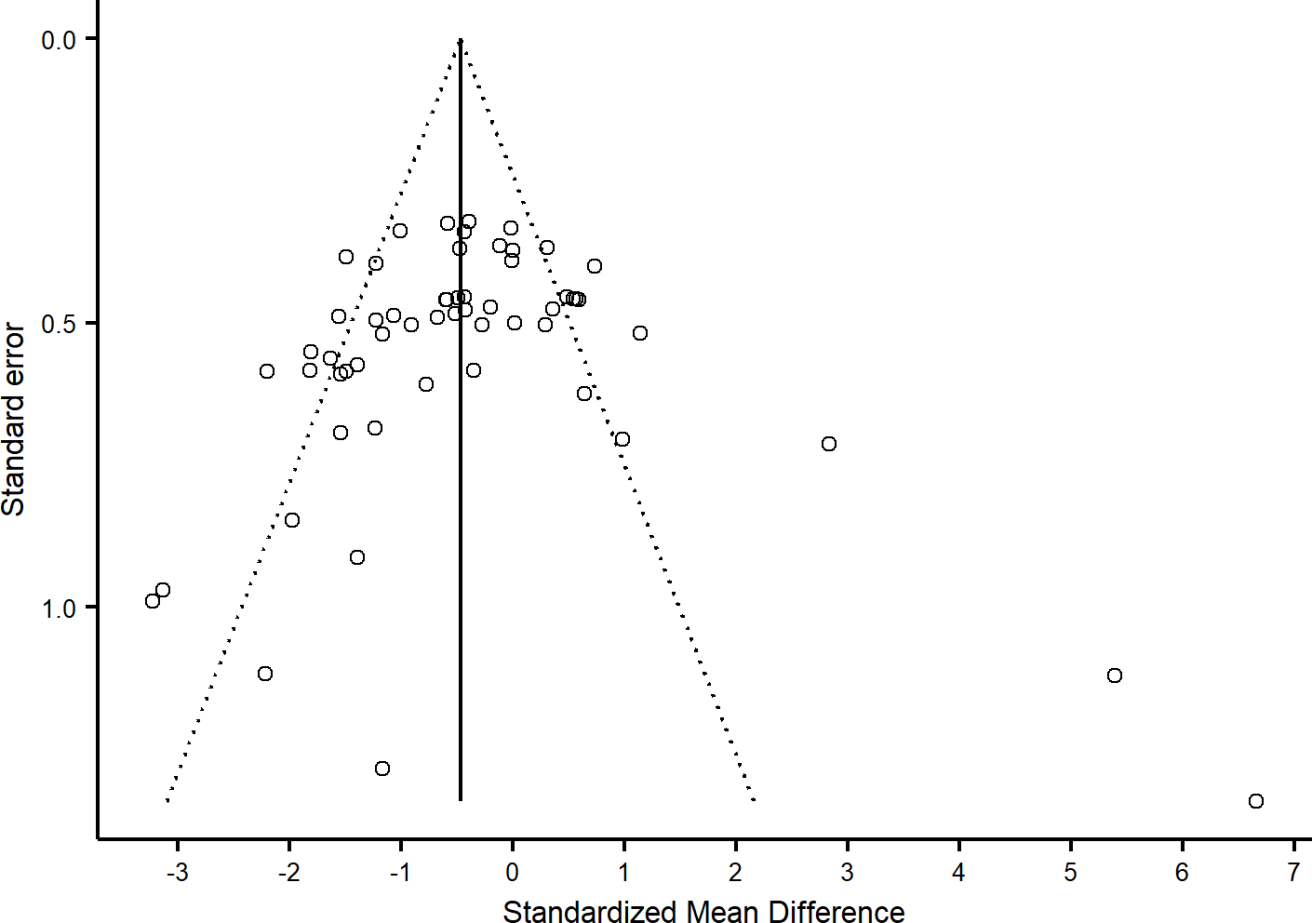
Funnel plot of preclinical standardized mean differences.

Visual inspection of a funnel plot (Fig 4) and the use of an Egger test (*t* = -0.15, *df* = 55, *p* = 0.880) did not suggest the presence of publication bias, although several SMDs fell outside of the expected area of the funnel plot. Factors other than publication bias can contribute to funnel plot asymmetry, including heterogeneity and other forms of bias [66]. There was moderate heterogeneity across the 33 experimental groups (I^2^ = 70.5%), indicating that 70.5% of the variation between study outcomes is attributable to inconsistency between the studies. Funnel plot asymmetry and heterogeneity are well-documented problems present in meta-analyses of animal research [67]. Factors such as subject species/strain, sample size, and additional experimental conditions can contribute to these issues, although inclusion of study characteristics as moderating variables and subgroup analyses did not reduce heterogeneity in the present analysis.

### Clinical meta-analysis

#### Study selection and characteristics

Fourteen studies [34, 41, 68–79], consisting of 1527 individuals, were included in the clinical meta-analysis (Fig 2 and Table 2). Eight studies assessed the effect of probiotic administration on healthy samples, while six studies did so with clinical samples. Of the six studies that used clinical participants, four studies investigated participants with irritable bowel syndrome [69, 70, 72, 74], one study investigated participants with at least moderate mood disturbance [73], and one study investigated participants with cancer [79]. Two studies divided their participants receiving probiotic into low and high dose groups [69, 70]. One study used a crossover randomized controlled trial design [41], while the other 13 studies used a parallel design. Species from the *Lactobacillus*, *Bifidobacterium*, *Pediococcus*, *Streptococcus*, and *Clostridium* genera were used as probiotics.

#### Bias assessment

The assessment for the included studies’ risk of bias can be viewed in Table 4. All 14 studies had a low risk of attrition bias, and most studies had a low risk of both reporting and other bias. Less than half of the studies included details regarding allocation concealment. Overall, the risk of bias for each included study ranged from low to unclear.

**Table 4:** Clinical risk of bias assessment.

Study	Random sequence generation	Allocation concealment	Blinding of participants	Blinding of assessor	Incomplete outcome data	Selective reporting	Other bias
Kato-Kataoka et al. 2016	Unclear	Unclear	Unclear	Unclear	Low	Low	Low
Kelly et al. 2017	Unclear	Unclear	Unclear	Unclear	Low	Unclear	Unclear
Lorenzo-Zuniga et al. 2014	Low	Unclear	Low	Low	Low	Low	Low
Lyra et al. 2016	Low	Unclear	Low	Low	Low	Low	Low
Marcos et al. 2004	Unclear	Unclear	Unclear	Unclear	Low	Low	Unclear
Messaoudi et al. 2011	Low	Low	Low	Low	Low	Unclear	Low
Pinto-Sanchez et al.	Low	Low	Low	Low	Low	Low	Low
Romijn et al. 2017	Low	Low	Low	Low	Low	Low	Low
Simren et al. 2010	Unclear	Low	Low	Low	Low	Low	Low
Slykerman et al. 2015	Unclear	Unclear	Low	Low	Low	Low	Low
Steenbergen et al. 2015	Unclear	Unclear	Low	Low	Low	Low	Low
Takada et al. 2016	Low	Unclear	Unclear	Unclear	Low	Low	Low
Takada et al. 2017	Low	Low	Unclear	Unclear	Low	Low	Low
Yang et al. 2016	Unclear	Unclear	Unclear	Unclear	Low	Low	Unclear

#### Probiotic efficacy

**Fig 5:**
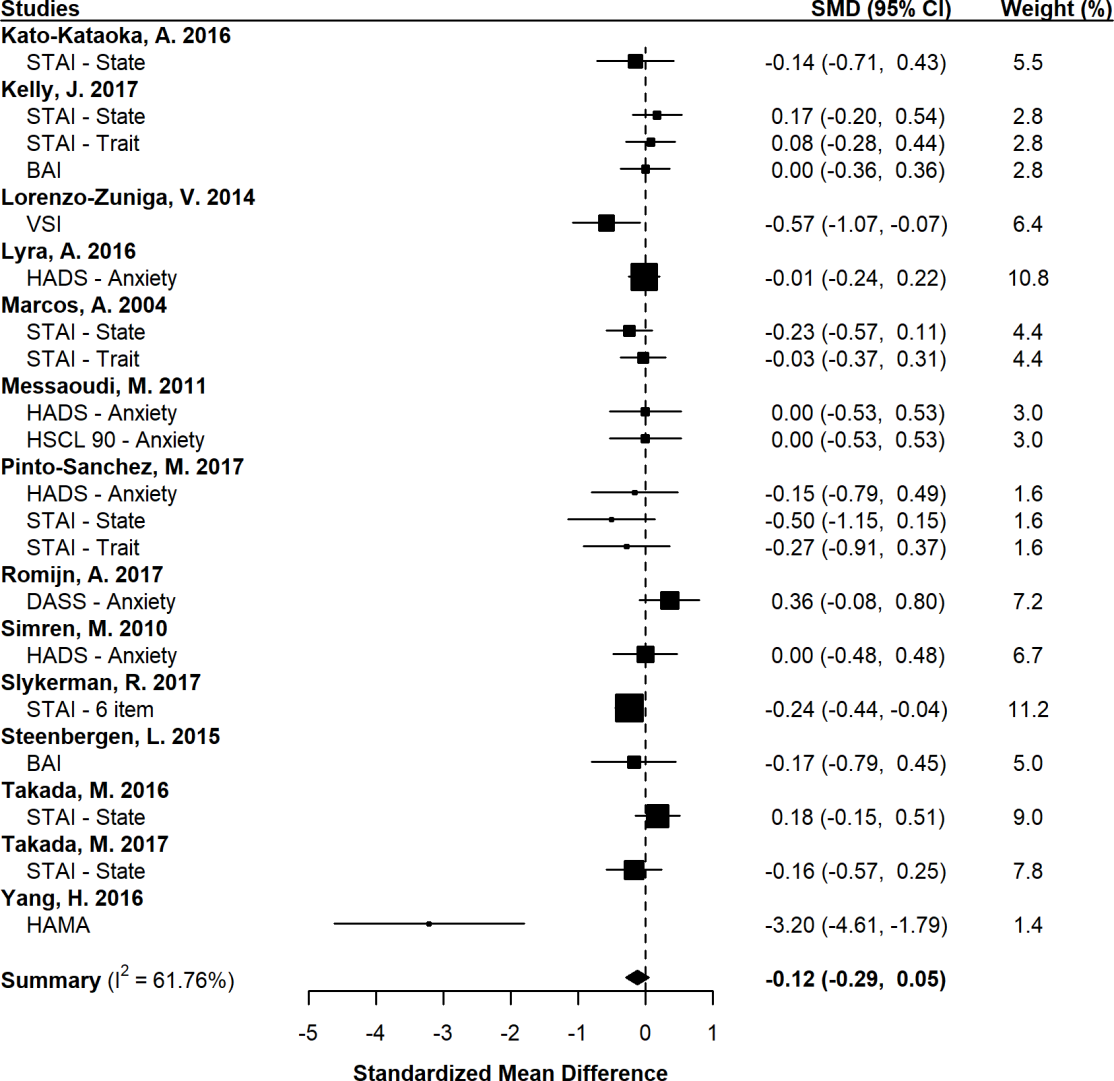
Forest plot of clinical studies investigating the effect of probiotics on anxiety-like behavior. SMD = Standardized mean difference; CI = Confidence interval; STAI = State-Trait Anxiety Inventory; BAI = Beck Anxiety Inventory; VSI = Visceral Sensitivity Index; HADS = Hospital Anxiety and Depression Scale; HSCL-90 = Hopkins Symptom Checklist-90; DASS = Depression Anxiety Stress Scales; HAM-A = Hamilton Anxiety Rating Scale.

Combining standardized mean differences (SMDs) for the 14 included studies revealed a pooled SMD of -0.12 (95% CI: -0.29–0.05, *p* = 0.151; Fig 5), indicating that probiotic administration did not result in a significant reduction of anxiety. Neither sample size (β = 0.00, 95% CI: 0.00–0.00, *p* = 0.746), probiotic duration (β = 0.00, 95% CI: -0.01–0.01, *p* = 0.915), nor probiotic dose (β = 0.00, 95% CI: -0.01–0.01, *p* = 0.433) provided a moderating influence. Additionally, subgroup analyses revealed that probiotic administration did not result in a significant reduction of anxiety in healthy (SMD = -0.10, 95% CI: -0.33–0.13, *p* = 0.283) or clinical participants (SMD = -0.33, 95% CI: -1.08–0.43, *p* = 0.312).

#### Publication bias and heterogeneity

**Fig 6:**
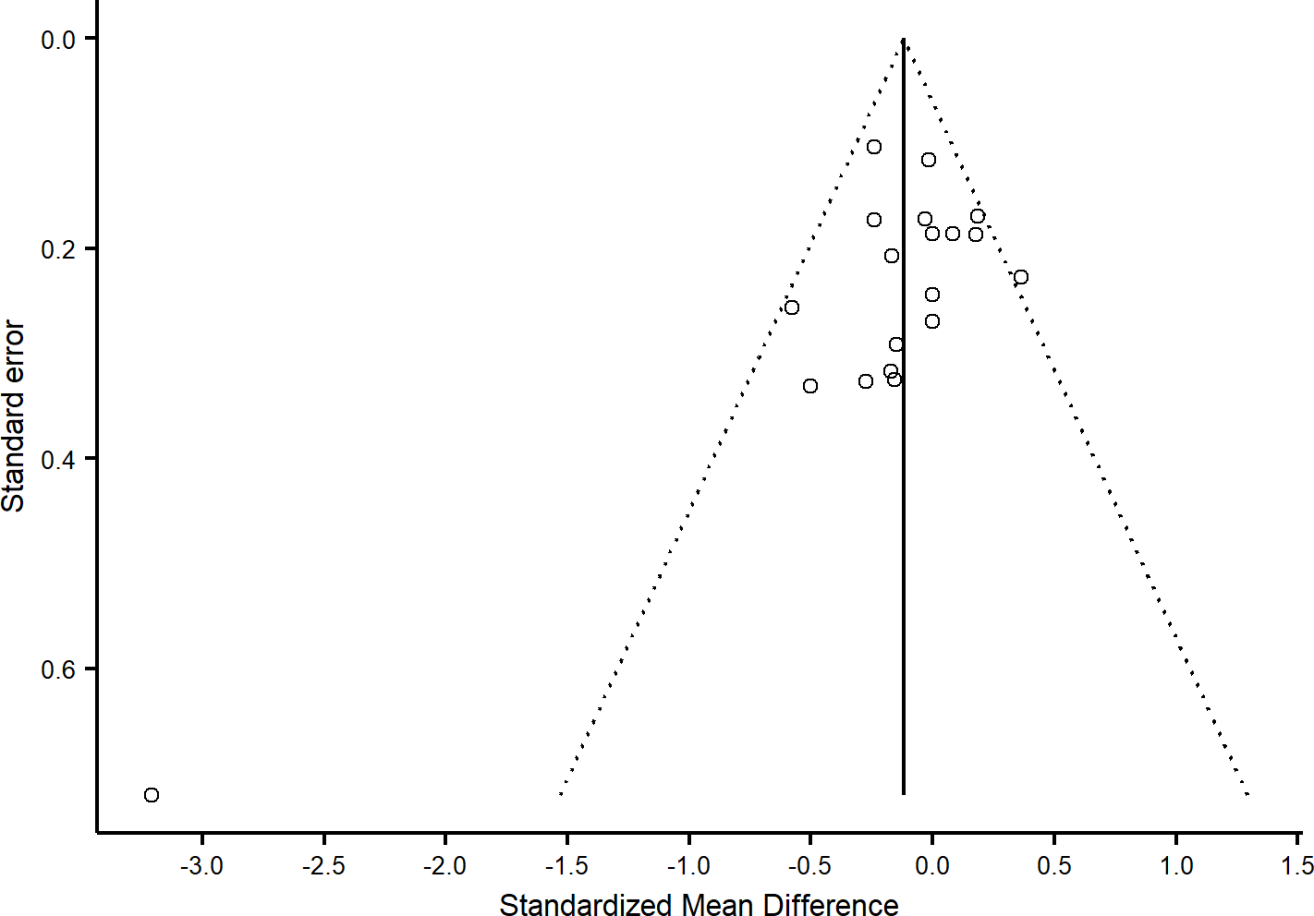
Funnel plot of clinical standardized mean differences.

Visual inspection of Fig 6 demonstrated symmetry, apart from one study [79], while the use of an Egger test similarly did not suggest the presence of publication bias (*t* = 1.52, *df* = 18, *p* = 0.146). There was also moderate heterogeneity across the 14 included studies (I^2^ = 61.8%). Removal of the study by Yang et al. [79] resulted in low heterogeneity (I^2^ = 21.7%); however, the results of the meta-analysis remained unchanged.

## Discussion

The present systematic review and meta-analysis of 22 preclinical studies (743 animals) revealed a significant overall effect of probiotic administration in reducing anxiety-like behavior in rodents. The observed pooled standardized mean difference (SMD) of -0.47 reflects a medium-sized effect of probiotic interventions in comparison with non-probiotic controls. At the level of individual trials, 12 of the 22 included animal studies found that probiotics significantly reduced anxiety-like behavior on at least one outcome measure, with the remaining 10 studies finding either no effect or (in one case) *increased* anxiety-like behavior.

In contrast, only 3 of the 14 included clinical studies (encompassing 1527 individuals) found that probiotics significantly reduced symptoms of anxiety. Notably, probiotics also yielded no overall anxiolytic effects in the present meta-analysis. Subgroup analyses likewise observed no significant probiotic effects among either healthy or diseased human participants. These findings stand in stark contrast with the conclusions of two recent qualitative reviews, both of which proposed that probiotics may have anxiolytic properties [27, 29]. However, unlike such reviews, the present study utilized meta-analytic techniques to quantitatively evaluate the magnitude of probiotic effects on anxiety, as well as the degree to which the reported effects of different studies varied. This approach also enabled the inclusion of multiple measures of anxiety from relevant studies in a single summary analysis. As such, the reported results provide the only comprehensive review to date of the relevant research.

In perhaps this study’s most important set of subgroup analyses, probiotics were found to successfully reduce anxiety-like behavior in diseased, but not in healthy, rodents. Although the utilized disease models varied considerably across studies—ranging from rodents with induced intestinal inflammation [47, 48, 53] to those exposed to stressful conditions [49, 54, 59] to those fed an unhealthy diet [44, 46]—they all reflected the presence of a pathological state that might be associated with elevated stress or anxious arousal. It is possible, therefore, that the anxiolytic effects of probiotics only occur above a baseline threshold level of heightened anxious arousal. If so, this phenomenon could help explain the absence of an observed therapeutic (anti-anxiety) effect of probiotic supplementation in our meta-analysis of human trials, inasmuch as none of the included studies specifically recruited participants on the basis of anxiety-related symptomatology. Indeed, the majority (8) of included studies simply assessed the effect of probiotics on self-reported anxiety levels among healthy, non-clinical participants. Another four studies recruited patients with irritable bowel syndrome (IBS) [69, 70, 72, 74], and although such patients may sometimes experience elevated anxiety [13], it is not a defining feature of the disorder. The remaining two clinical studies likewise selected participants based on criteria other than anxiety severity [73, 79]. Simply put: the general absence of clinically salient anxiety among human participants in the extant probiotic literature may have obscured any potential anxiolytic effects. Accordingly, it will be useful and informative for future investigations in this area to explicitly target participants characterized by clinically significant anxiety.

The discrepancy between preclinical and clinical studies may also be due to differences in the way that anxiety was evaluated across these groups. Whereas anxiety in humans was assessed entirely through self-report measures, anxiety in rodents was assessed through behavioral observations. There is evidence that, during the treatment of emotion-based disorders such as depression or anxiety, cognitive and behavioral processes improve prior to any subjective awareness of recovery, which may take weeks to be fully realized [80]. It is possible that self-report questionnaires of anxiety are not sensitive enough to detect probiotic-induced anxiolysis, at least under typically studied treatment durations. Only half of the included clinical studies administered probiotics for at least eight weeks, which is often used as an upper cutoff when determining response to pharmacological treatments such as antidepressants. In addition to longer treatment durations, alternative forms of assessment, such as functional imaging techniques, may be needed in clinical studies to accurately measure the anxiolytic impact of probiotics. Encouragingly, at least one study to date has demonstrated that probiotics can alter emotional processing as measured by functional imaging [23].

In terms of the specific probiotic supplement regimens employed, the 14 relevant human trials to date were characterized by such a high level of between-study heterogeneity that more fine-grained analyses of individual probiotic species were precluded. However, the preclinical studies were subjected to further subgroup analyses on the basis of four species of probiotic that were each utilized in several studies. Among these four candidate species, only *Lactobacillus* (*L*.) *rhamnosus* was found to significantly reduce anxiety-like behavior, with a rather large standardized mean difference (SMD) of -0.77 across the relevant trials [49, 50, 52]. Each of the *L*. *rhamnosus* studies used an administration duration of 28 days, with specific strains consisting of *L*. *rhamnosus* JB-1 [49, 50] and *L*. *rhamnosus* GG MTCC 1408 [52]. Notably, *L*. *rhamnosus* was also the probiotic species used in one of the only studies to observe a significant anxiolytic effect in humans [75]. This particular species has been widely investigated, and has been shown to attenuate the symptoms of various gastrointestinal and allergic diseases [81]. Based on our results, it appears that *L*. *rhamnosus* may also have psychotropic properties, and should be further and more extensively investigated for its anxiolytic potential.

Other probiotic species that were shown to significantly reduce anxiety-like behavior in individual rodent studies include *L*. *helveticus* [55, 58], *Bifidobacterium* (*B*.) *longum* [47], *B*. *adolescentis* [54], and combined *L*. *rhamnosus* and *B*. *longum* [48]. Conversely, one species of probiotic was shown to *increase* anxiety-like behavior in rodents: *L*. *casei* [45]. While *Lactobacillus* and *Bifidobacterium* species of bacteria are the most commonly investigated probiotics [82], the different species are not identical, and even strains within the same species can have unique effects on the body [83]. Because of this, candidate probiotics require extensive study and characterization prior to clinical application.

Notably, the dose-response curve of probiotics also remains almost completely uninvestigated when it comes to their potential psychoactive effects. In fact, the weight-adjusted probiotic dosages (colony-forming units per gram of body weight) used in the rodent trials reviewed herein were typically *hundreds of times larger* than the corresponding dosages used in the human trials. And this fact raises the possibility that the significant anxiolytic effect of supplementation in the rodent meta-analysis and the null effect in the human meta-analysis both reflect—at least in part—the much higher dosing schedule utilized with the rodents. The same consideration could also help explain the more robust anxiolytic effect observed among mice versus rats, as the mice typically received higher weight-adjusted probiotic doses by virtue of being about 10 times smaller than rats, on average. By extension, it is conceivable that future investigators could discover the most effectively anxiolytic probiotic dosages in humans to be dozens—or perhaps even hundreds—of times higher than those employed to date.

It is possible that probiotics reduce anxiety-like behavior by influencing the immune system, which is a primary component of the microbiota-gut-brain axis [10]. Many of the included preclinical studies found the anxiolytic effects of probiotics to be accompanied by beneficial alterations in immune functioning [49, 52, 54, 55, 57, 58]. And the microbiota continuously stimulates a complex and dynamic immune response by interacting with the intestinal barrier [84]. Germ-free mice without a microbiota have an impaired immune response [85], and induced dysbiosis has been linked with inflammatory bowel disease [86]. As such, probiotics may improve mental health by restoring microbiota-mediated immune activation to an adaptive level. Another possibility is that the anxiolytic effects observed herein were due to an alteration of activity in the vagus nerve, also a major connection between the microbiota and the brain [10]. In fact, two of the reviewed preclinical studies found that inhibition of the vagus nerve—a major pathway between the microbiota and the central nervous system [87]—prevented probiotic-induced reductions of anxiety-like behavior [47, 50].

It should be emphasized that none of the analyzed preclinical studies provided sufficient detail regarding risk of selection, performance, or detection biases, making it so that the overall risk of bias for each preclinical study was unclear. Most clinical studies were similarly rated as having an unclear risk of bias, especially within the domains of randomization, allocation concealment, and blinding. As such, there is some concern that bias present in the included studies may be affecting the observed results. Failure to account for potential biases has been shown to influence study outcomes and can lead to overestimation of observed effects [88, 89]. For example, animal studies that do not employ randomization procedures have been found to have significantly higher standardized mean differences than those that do [90]. Furthermore, an incomplete description of study methods can complicate replication efforts and evaluations of study reliability. Poor reporting of bias risk criteria is a particular issue within the broader preclinical literature, and certainly not isolated to probiotic research [91]. The use of appropriate randomization, allocation concealment, and blinding procedures, as well as proper reporting thereof, will greatly aid preclinical and clinical probiotic development research.

Two of the 14 included clinical studies found that probiotic administration resulted in a significant improvement of anxiety, compared to placebo, without contributing substantially to statistical heterogeneity [69, 75]. Notably, Slykerman et al. [75] administered probiotics to pregnant women for up to one year, the longest duration of any study included in this analysis. Because the composition of the microbiota is relatively stable and at least somewhat resistant to change induced by external disturbances, including probiotics [92]—and in light of evidence that probiotics may only exert transient effects on the body [93, 94]—it is possible that long-term probiotic supplementation is necessary to significantly alter the microbiota composition and induce beneficial changes in psychological functioning. It may also be that pregnancy represents a unique window for probiotic-based intervention, as there is evidence that the microbiota changes drastically during pregnancy [95]. However, the variability of the microbiota during pregnancy remains unknown and a recent longitudinal study by DiGiulio et al. [96] found that the microbiotas of pregnant women are stable across time.

Lorenzo-Zuniga et al. [69] used a measure of gastrointestinal-specific anxiety—the Visceral Sensitivity Index (VSI) [97]—in adults with irritable bowel syndrome (IBS). While the VSI does assess general anxiety, as evidenced by its strong convergent validity with other measures of general anxiety, it also captures anxiety specific to gastrointestinal (GI) symptom severity [97, 98]. Given this finding, it may be that probiotics are more effective at alleviating GI-specific anxiety than general anxiety, at least in individuals with abnormal GI functioning. Probiotics specifically interact with the GI tract and appear to be well-suited for the treatment of GI distress. Indeed, a recent systematic review and meta-analysis has found that probiotics successfully reduce GI symptom severity in individuals with IBS while improving the integrity of the intestinal barrier [20]. However, it may be that probiotic-induced reductions in GI-specific anxiety are attributable to a reduction in GI symptoms, as Lorenzo-Zuniga et al. [69] found that GI-related quality of life improved prior to GI-specific anxiety. Another possibility is that probiotics are more effective at reducing anxiety in individuals with GI dysfunction, due to the presence of a more impaired microbiota; however, the other three clinical studies in this analysis that selected subjects with IBS found that probiotics had no effect on general anxiety [70, 72, 74].

### Limitations

One important limitation of this study was the substantial heterogeneity present among both preclinical and clinical studies reviewed. Subject characteristics, outcome measures, probiotic strain (including single versus multispecies preparations), probiotic dose, and probiotic duration all varied substantially from study to study, and such variation likely contributed to the observed high level of statistical heterogeneity, which is problematic as high heterogeneity reduces the predictive validity of meta-analyses [99]. Although the use of random-effects models, as done here, can help to account for such heterogeneity [99], appropriate caution still needs to be taken when interpreting the present results, as they may not accurately reflect the true effect of probiotics.

Another limitation was that only 14 studies were included in the clinical meta-analysis, due to a lack of relevant research attributable to the novelty of using probiotics as a psychotropic intervention. The presence of more human trials could provide greater insight into the anxiolytic potential of probiotics and would also allow for more nuanced subgroup analyses. Additional preclinical research is similarly needed, as each preclinical subgroup analysis often encompassed only a handful of relevant standardized mean differences. A third limitation is that preclinical sample sizes (typically ranging from 10–40 animals) tended to be much smaller than those found in clinical studies. Although publication bias—a major concern when it comes to small sample size—was not observed among the included preclinical studies, small sample sizes may exacerbate other biases that can negatively affect the reliability and validity of study outcomes [100]. This may help explain the differential impact of probiotics observed in preclinical and clinical studies.

### Conclusion

While probiotic administration reduces anxiety-like behavior in rodents, the current state of clinical research does not (yet) support probiotics as an efficacious treatment for anxiety. *Lactobacillus rhamnosus* was nonetheless identified as a candidate anxiolytic probiotic species by both preclinical and clinical studies. An important target of future clinical investigation is the examination of the impact of probiotics on clinically significant anxiety, as probiotics only significantly reduced anxiety-like behavior in diseased rodents. It may also be worthwhile to investigate both higher dosages and longer durations of probiotic administration, as well as the effect of probiotics on specific subtypes of anxiety, such as anxiety related to gastrointestinal distress.

## Supporting information

S1 Appendix Preclinical and clinical PubMed search algorithm. (DOCX)

S2 Appendix Variable dictionary. (DOCX)

S1 Fig Full forest plot of preclinical studies. SMD = Standardized mean difference; CI = Confidence interval; EPM = Elevated plus maze; LDT = Light-dark test; Step-down = Step-down test; OFT = Open field test.
(PDF)

S1 Data Preclinical data. (CSV)

S2 Data Clinical data. (CSV)

S1 Text PRISMA checklist. (DOC)

## References

[1] Kessler RC, Aguilar-Gaxiola S, Alonso J, Chatterji S, Lee S, Ormel J, et al The global burden of mental disorders: an update from the WHO World Mental Health (WMH) surveys. Epidemiol Psichiatr Soc. 2009;18(1): 23–33. Epub 2009/04/22. ; PubMed Central PMCID: PMCPMC3039289. 19378696 10.1017/s1121189x00001421 PMC3039289

[2] Baxter AJ, Vos T, Scott KM, Ferrari AJ, Whiteford HA. The global burden of anxiety disorders in 2010. Psychol Med. 2014;44(11): 2363–74. Epub 2014/01/24. doi: 10.1017/S0033291713003243 . 24451993 10.1017/S0033291713003243

[3] Roest AM, Martens EJ, de Jonge P, Denollet J. Anxiety and risk of incident coronary heart disease: a meta-analysis. J Am Coll Cardiol. 2010;56(1): 38–46. Epub 2010/07/14. doi: 10.1016/j.jacc.2010.03.034 . 20620715 10.1016/j.jacc.2010.03.034

[4] Cox RC, Olatunji BO. A systematic review of sleep disturbance in anxiety and related disorders. J Anxiety Disord. 2016;37: 104–29. Epub 2016/01/09. doi: 10.1016/j.janxdis.2015.12.001 . 26745517 10.1016/j.janxdis.2015.12.001

[5] Lai HM, Cleary M, Sitharthan T, Hunt GE. Prevalence of comorbid substance use, anxiety and mood disorders in epidemiological surveys, 1990–2014: A systematic review and meta-analysis. Drug Alcohol Depend. 2015;154: 1–13. Epub 2015/06/15. doi: 10.1016/j.drugalcdep.2015.05.031 . 26072219 10.1016/j.drugalcdep.2015.05.031

[6] Bandelow B, Reitt M, Rover C, Michaelis S, Gorlich Y, Wedekind D. Efficacy of treatments for anxiety disorders: A meta-analysis. Int Clin Psychopharmacol. 2015;30(4): 183–92. Epub 2015/05/02. doi: 10.1097/YIC.0000000000000078 . 25932596 10.1097/YIC.0000000000000078

[7] Loerinc AG, Meuret AE, Twohig MP, Rosenfield D, Bluett EJ, Craske MG. Response rates for CBT for anxiety disorders: Need for standardized criteria. Clin Psychol Rev. 2015;42: 72–82. Epub 2015/09/01. doi: 10.1016/j.cpr.2015.08.004 . 26319194 10.1016/j.cpr.2015.08.004

[8] Baldwin D, Woods R, Lawson R, Taylor D. Efficacy of drug treatments for generalised anxiety disorder: Systematic review and meta-analysis. BMJ. 2011;342: d1199 Epub 2011/03/15. doi: 10.1136/bmj.d1199 . 21398351 10.1136/bmj.d1199

[9] Backhed F, Ley RE, Sonnenburg JL, Peterson DA, Gordon JI. Host-bacterial mutualism in the human intestine. Science. 2005;307(5717): 1915–20. Epub 2005/03/26. doi: 10.1126/science.1104816 . 15790844 10.1126/science.1104816

[10] Carabotti M, Scirocco A, Maselli MA, Severi C. The gut-brain axis: Interactions between enteric microbiota, central and enteric nervous systems. Annals of Gastroenterology. 2015;28(2): 203–9. PubMed PMID: WOS:000359016600007. 25830558 PMC4367209

[11] de Vos WM, de Vos EA. Role of the intestinal microbiome in health and disease: From correlation to causation. Nutr Rev. 2012;70 Suppl 1: S45–56. Epub 2012/08/17. doi: 10.1111/j.1753-4887.2012.00505.x . 22861807 10.1111/j.1753-4887.2012.00505.x

[12] Walter SA, Jones MP, Talley NJ, Kjellstrom L, Nyhlin H, Andreasson AN, et al Abdominal pain is associated with anxiety and depression scores in a sample of the general adult population with no signs of organic gastrointestinal disease. Neurogastroenterol Motil. 2013;25(9): 741–e576. Epub 2013/05/23. doi: 10.1111/nmo.12155 . 23692044 10.1111/nmo.12155

[13] Fond G, Loundou A, Hamdani N, Boukouaci W, Dargel A, Oliveira J, et al Anxiety and depression comorbidities in irritable bowel syndrome (IBS): A systematic review and meta-analysis. Eur Arch Psychiatry Clin Neurosci. 2014;264(8): 651–60. Epub 2014/04/08. doi: 10.1007/s00406-014-0502-z . 24705634 10.1007/s00406-014-0502-z

[14] Neuendorf R, Harding A, Stello N, Hanes D, Wahbeh H. Depression and anxiety in patients with Inflammatory Bowel Disease: A systematic review. J Psychosom Res. 2016;87: 70–80. Epub 2016/07/15. doi: 10.1016/j.jpsychores.2016.06.001 . 27411754 10.1016/j.jpsychores.2016.06.001

[15] Jernberg C, Lofmark S, Edlund C, Jansson JK. Long-term ecological impacts of antibiotic administration on the human intestinal microbiota. Isme j. 2007;1(1): 56–66. Epub 2007/11/29. doi: 10.1038/ismej.2007.3 . 18043614 10.1038/ismej.2007.3

[16] Lurie I, Yang YX, Haynes K, Mamtani R, Boursi B. Antibiotic exposure and the risk for depression, anxiety, or psychosis: a nested case-control study. J Clin Psychiatry. 2015;76(11): 1522–8. Epub 2015/11/19. doi: 10.4088/JCP.15m09961 . 26580313 10.4088/JCP.15m09961

[17] Bruch JD. Intestinal infection associated with future onset of an anxiety disorder: Results of a nationally representative study. Brain Behav Immun. 2016;57: 222–6. Epub 2016/05/26. doi: 10.1016/j.bbi.2016.05.014 . 27223096 10.1016/j.bbi.2016.05.014

[18] Goehler LE, Park SM, Opitz N, Lyte M, Gaykema RP. Campylobacter jejuni infection increases anxiety-like behavior in the holeboard: possible anatomical substrates for viscerosensory modulation of exploratory behavior. Brain Behav Immun. 2008;22(3): 354–66. Epub 2007/10/09. doi: 10.1016/j.bbi.2007.08.009 ; PubMed Central PMCID: PMCPMC2259293. 17920243 10.1016/j.bbi.2007.08.009 PMC2259293

[19] Lyte M, Varcoe JJ, Bailey MT. Anxiogenic effect of subclinical bacterial infection in mice in the absence of overt immune activation. Physiol Behav. 1998;65(1): 63–8. Epub 1998/11/12. . 9811366 10.1016/s0031-9384(98)00145-0

[20] Didari T, Mozaffari S, Nikfar S, Abdollahi M. Effectiveness of probiotics in irritable bowel syndrome: Updated systematic review with meta-analysis. World J Gastroenterol. 2015;21(10): 3072–84. Epub 2015/03/18. doi: 10.3748/wjg.v21.i10.3072 ; PubMed Central PMCID: PMCPMC4356930. 25780308 10.3748/wjg.v21.i10.3072 PMC4356930

[21] Ganji-Arjenaki M, Rafieian-Kopaei M. Probiotics are a good choice in remission of inflammatory bowel diseases: A meta analysis and systematic review. J Cell Physiol. 2017 Epub 2017/03/16. doi: 10.1002/jcp.25911 . 28294322 10.1002/jcp.25911

[22] Mantegazza C, Molinari P, D'Auria E, Sonnino M, Morelli L, Zuccotti GV. Probiotics and antibiotic-associated diarrhea in children: A review and new evidence on Lactobacillus rhamnosus GG during and after antibiotic treatment. Pharmacol Res. 2017 Epub 2017/08/23. doi: 10.1016/j.phrs.2017.08.001 . 28827186 10.1016/j.phrs.2017.08.001

[23] Tillisch K, Labus J, Kilpatrick L, Jiang Z, Stains J, Ebrat B, et al Consumption of fermented milk product with probiotic modulates brain activity. Gastroenterology. 2013;144(7): 1394–401, 401.e1-4. Epub 2013/03/12. doi: 10.1053/j.gastro.2013.02.043 ; PubMed Central PMCID: PMCPMC3839572. 23474283 10.1053/j.gastro.2013.02.043 PMC3839572

[24] Benton D, Williams C, Brown A. Impact of consuming a milk drink containing a probiotic on mood and cognition. Eur J Clin Nutr. 2007;61(3): 355–61. Epub 2006/12/08. doi: 10.1038/sj.ejcn.1602546 . 17151594 10.1038/sj.ejcn.1602546

[25] Ait-Belgnaoui A, Durand H, Cartier C, Chaumaz G, Eutamene H, Ferrier L, et al Prevention of gut leakiness by a probiotic treatment leads to attenuated HPA response to an acute psychological stress in rats. Psychoneuroendocrinology. 2012;37(11): 1885–95. Epub 2012/05/01. doi: 10.1016/j.psyneuen.2012.03.024 . 22541937 10.1016/j.psyneuen.2012.03.024

[26] Ait-Belgnaoui A, Colom A, Braniste V, Ramalho L, Marrot A, Cartier C, et al Probiotic gut effect prevents the chronic psychological stress-induced brain activity abnormality in mice. Neurogastroenterol Motil. 2014;26(4): 510–20. Epub 2014/01/01. doi: 10.1111/nmo.12295 . 24372793 10.1111/nmo.12295

[27] Pirbaglou M, Katz J, de Souza RJ, Stearns JC, Motamed M, Ritvo P. Probiotic supplementation can positively affect anxiety and depressive symptoms: a systematic review of randomized controlled trials. Nutr Res. 2016;36(9): 889–98. Epub 2016/09/17. doi: 10.1016/j.nutres.2016.06.009 . 27632908 10.1016/j.nutres.2016.06.009

[28] Romijn AR, Rucklidge JJ. Systematic review of evidence to support the theory of psychobiotics. Nutr Rev. 2015;73(10): 675–93. Epub 2015/09/16. doi: 10.1093/nutrit/nuv025 . 26370263 10.1093/nutrit/nuv025

[29] Wang H, Lee IS, Braun C, Enck P. Effect of Probiotics on Central Nervous System Functions in Animals and Humans: A Systematic Review. J Neurogastroenterol Motil. 2016;22(4): 589–605. Epub 2016/07/15. doi: 10.5056/jnm16018 ; PubMed Central PMCID: PMCPMC5056568. 27413138 10.5056/jnm16018 PMC5056568

[30] Liberati A, Altman DG, Tetzlaff J, Mulrow C, Gotzsche PC, Ioannidis JP, et al The PRISMA statement for reporting systematic reviews and meta-analyses of studies that evaluate health care interventions: explanation and elaboration. PLoS Med. 2009;6(7): e1000100 Epub 2009/07/22. doi: 10.1371/journal.pmed.1000100 ; PubMed Central PMCID: PMCPMC2707010. 19621070 10.1371/journal.pmed.1000100 PMC2707010

[31] Vesterinen HM, Sena ES, Egan KJ, Hirst TC, Churolov L, Currie GL, et al Meta-analysis of data from animal studies: a practical guide. J Neurosci Methods. 2014;221: 92–102. Epub 2013/10/09. doi: 10.1016/j.jneumeth.2013.09.010 . 24099992 10.1016/j.jneumeth.2013.09.010

[32] Hooijmans CR, Rovers MM, de Vries RB, Leenaars M, Ritskes-Hoitinga M, Langendam MW. SYRCLE's risk of bias tool for animal studies. BMC Med Res Methodol. 2014;14: 43 Epub 2014/03/29. doi: 10.1186/1471-2288-14-43 ; PubMed Central PMCID: PMCPMC4230647. 24667063 10.1186/1471-2288-14-43 PMC4230647

[33] Higgins JP, Altman DG, Gotzsche PC, Juni P, Moher D, Oxman AD, et al The Cochrane Collaboration's tool for assessing risk of bias in randomised trials. BMJ. 2011;343: d5928 Epub 2011/10/20. doi: 10.1136/bmj.d5928 ; PubMed Central PMCID: PMCPMC3196245. 22008217 10.1136/bmj.d5928 PMC3196245

[34] Messaoudi M, Lalonde R, Violle N, Javelot H, Desor D, Nejdi A, et al Assessment of psychotropic-like properties of a probiotic formulation (Lactobacillus helveticus R0052 and Bifidobacterium longum R0175) in rats and human subjects. Br J Nutr. 2011;105(5): 755–64. Epub 2010/10/27. doi: 10.1017/S0007114510004319 . 20974015 10.1017/S0007114510004319

[35] Cochrane Handbook for Systematic Reviews of Interventions: The Cochrane Collaboration; 2011. Available from: http://training.cochrane.org/handbook.

[36] Rohatgi A. WebPlotDigitizer 2017. Available from: http://arohatgi.info/WebPlotDigitizer.

[37] Guyot P, Ades AE, Ouwens MJ, Welton NJ. Enhanced secondary analysis of survival data: reconstructing the data from published Kaplan-Meier survival curves. BMC Med Res Methodol. 2012;12: 9 Epub 2012/02/03. doi: 10.1186/1471-2288-12-9 ; PubMed Central PMCID: PMCPMC3313891. 22297116 10.1186/1471-2288-12-9 PMC3313891

[38] Tsafnat G, Glasziou P, Choong MK, Dunn A, Galgani F, Coiera E. Systematic review automation technologies. Syst Rev. 2014;3: 74 Epub 2014/07/10. doi: 10.1186/2046-4053-3-74 ; PubMed Central PMCID: PMCPMC4100748. 25005128 10.1186/2046-4053-3-74 PMC4100748

[39] R Core Team. R: A language and environment for statistical computing. Vienna, Austria: R Foundation for Statistical Computing; 2016.

[40] Tipton E. Small sample adjustments for robust variance estimation with meta-regression. Psychol Methods. 2015;20(3): 375–93. Epub 2014/04/30. doi: 10.1037/met0000011 . 24773356 10.1037/met0000011

[41] Kelly JR, Allen AP, Temko A, Hutch W, Kennedy PJ, Farid N, et al Lost in translation? The potential psychobiotic Lactobacillus rhamnosus (JB-1) fails to modulate stress or cognitive performance in healthy male subjects. Brain Behavior and Immunity. 2017;61: 50–9. doi: 10.1016/j.bbi.2016.11.018 PubMed PMID: WOS:000395365900008. 27865949 10.1016/j.bbi.2016.11.018

[42] Higgins JP, Thompson SG, Deeks JJ, Altman DG. Measuring inconsistency in meta-analyses. BMJ. 2003;327(7414): 557–60. Epub 2003/09/06. doi: 10.1136/bmj.327.7414.557 ; PubMed Central PMCID: PMCPMC192859. 12958120 10.1136/bmj.327.7414.557 PMC192859

[43] Egger M, Davey Smith G, Schneider M, Minder C. Bias in meta-analysis detected by a simple, graphical test. BMJ. 1997;315(7109): 629–34. Epub 1997/10/06. ; PubMed Central PMCID: PMCPMC2127453. 9310563 10.1136/bmj.315.7109.629 PMC2127453

[44] Agusti A, Moya-Perez A, Campillo I, Montserrat-de la Paz S, Cerrudo V, Perez-Villalba A, et al Bifidobacterium pseudocatenulatum CECT 7765 ameliorates neuroendocrine alterations associated with an exaggerated stress response and anhedonia in obese mice. Mol Neurobiol. 2017 Epub 2017/09/19. doi: 10.1007/s12035-017-0768-z . 28921462 10.1007/s12035-017-0768-z

[45] Barrera-Bugueno C, Realini O, Escobar-Luna J, Sotomayor-Zarate R, Gotteland M, Julio-Pieper M, et al Anxiogenic effects of a Lactobacillus, inulin and the synbiotic on healthy juvenile rats. Neuroscience. 2017;359: 18–29. Epub 2017/07/12. doi: 10.1016/j.neuroscience.2017.06.064 . 28694176 10.1016/j.neuroscience.2017.06.064

[46] Beilharz JE, Kaakoush NO, Maniam J, Morris MJ. Cafeteria diet and probiotic therapy: cross talk among memory, neuroplasticity, serotonin receptors and gut microbiota in the rat. Mol Psychiatry. 2017 Epub 2017/03/16. doi: 10.1038/mp.2017.38 . 28289278 10.1038/mp.2017.38

[47] Bercik P, Park AJ, Sinclair D, Khoshdel A, Lu J, Huang X, et al The anxiolytic effect of Bifidobacterium longum NCC3001 involves vagal pathways for gut-brain communication. Neurogastroenterol Motil. 2011;23(12): 1132–9. Epub 2011/10/13. doi: 10.1111/j.1365-2982.2011.01796.x ; PubMed Central PMCID: PMCPmc3413724. 21988661 10.1111/j.1365-2982.2011.01796.x PMC3413724

[48] Bercik P, Verdu EF, Foster JA, Macri J, Potter M, Huang X, et al Chronic gastrointestinal inflammation induces anxiety-like behavior and alters central nervous system biochemistry in mice. Gastroenterology. 2010;139(6): 2102–12.e1. Epub 2010/07/06. doi: 10.1053/j.gastro.2010.06.063 . 20600016 10.1053/j.gastro.2010.06.063

[49] Bharwani A, Mian MF, Surette MG, Bienenstock J, Forsythe P. Oral treatment with Lactobacillus rhamnosus attenuates behavioural deficits and immune changes in chronic social stress. BMC Med. 2017;15(1): 7 Epub 2017/01/12. doi: 10.1186/s12916-016-0771-7 ; PubMed Central PMCID: PMCPMC5225647. 28073366 10.1186/s12916-016-0771-7 PMC5225647

[50] Bravo JA, Forsythe P, Chew MV, Escaravage E, Savignac HM, Dinan TG, et al Ingestion of Lactobacillus strain regulates emotional behavior and central GABA receptor expression in a mouse via the vagus nerve. Proc Natl Acad Sci U S A. 2011;108(38): 16050–5. Epub 2011/08/31. doi: 10.1073/pnas.1102999108 ; PubMed Central PMCID: PMCPmc3179073. 21876150 10.1073/pnas.1102999108 PMC3179073

[51] Cowan CS, Callaghan BL, Richardson R. The effects of a probiotic formulation (Lactobacillus rhamnosus and L. helveticus) on developmental trajectories of emotional learning in stressed infant rats. Transl Psychiatry. 2016;6(5): e823 Epub 2016/06/01. doi: 10.1038/tp.2016.94 . 27244232 10.1038/tp.2016.94 PMC5545650

[52] Divyashri G, Krishna G, Muralidhara, Prapulla SG. Probiotic attributes, antioxidant, anti-inflammatory and neuromodulatory effects of Enterococcus faecium CFR 3003: In vitro and in vivo evidence. J Med Microbiol. 2015;64(12): 1527–40. Epub 2015/10/10. doi: 10.1099/jmm.0.000184 . 26450608 10.1099/jmm.0.000184

[53] Emge JR, Huynh K, Miller EN, Kaur M, Reardon C, Barrett KE, et al Modulation of the microbiota-gut-brain axis by probiotics in a murine model of inflammatory bowel disease. Am J Physiol Gastrointest Liver Physiol. 2016;310(11): G989–98. Epub 2016/04/09. doi: 10.1152/ajpgi.00086.2016 . 27056723 10.1152/ajpgi.00086.2016

[54] Jang HM, Jang SE, Han MJ, Kim DH. Anxiolytic-like effect of Bifidobacterium adolescentis IM38 in mice with or without immobilisation stress. Benef Microbes. 2017: 1–10. Epub 2017/10/04. doi: 10.3920/bm2016.0226 . 28969445 10.3920/BM2016.0226

[55] Liang S, Wang T, Hu X, Luo J, Li W, Wu X, et al Administration of Lactobacillus helveticus NS8 improves behavioral, cognitive, and biochemical aberrations caused by chronic restraint stress. Neuroscience. 2015;310: 561–77. Epub 2015/09/27. doi: 10.1016/j.neuroscience.2015.09.033 . 26408987 10.1016/j.neuroscience.2015.09.033

[56] Liu WH, Chuang HL, Huang YT, Wu CC, Chou GT, Wang S, et al Alteration of behavior and monoamine levels attributable to Lactobacillus plantarum PS128 in germ-free mice. Behav Brain Res. 2016;298(Pt B): 202–9. Epub 2015/11/03. doi: 10.1016/j.bbr.2015.10.046 . 26522841 10.1016/j.bbr.2015.10.046

[57] Liu YW, Liu WH, Wu CC, Juan YC, Wu YC, Tsai HP, et al Psychotropic effects of Lactobacillus plantarum PS128 in early life-stressed and naive adult mice. Brain Res. 2016;1631: 1–12. Epub 2015/12/02. doi: 10.1016/j.brainres.2015.11.018 . 26620542 10.1016/j.brainres.2015.11.018

[58] Luo J, Wang T, Liang S, Hu X, Li W, Jin F. Ingestion of Lactobacillus strain reduces anxiety and improves cognitive function in the hyperammonemia rat. Sci China Life Sci. 2014;57(3): 327–35. Epub 2014/02/21. doi: 10.1007/s11427-014-4615-4 . 24554471 10.1007/s11427-014-4615-4

[59] Mackos AR, Eubank TD, Parry NM, Bailey MT. Probiotic Lactobacillus reuteri attenuates the stressor-enhanced severity of Citrobacter rodentium infection. Infect Immun. 2013;81(9): 3253–63. Epub 2013/06/27. doi: 10.1128/IAI.00278-13 ; PubMed Central PMCID: PMCPMC3754198. 23798531 10.1128/IAI.00278-13 PMC3754198

[60] Matthews DM, Jenks SM. Ingestion of Mycobacterium vaccae decreases anxiety-related behavior and improves learning in mice. Behav Processes. 2013;96: 27–35. Epub 2013/03/05. doi: 10.1016/j.beproc.2013.02.007 . 23454729 10.1016/j.beproc.2013.02.007

[61] McKernan DP, Fitzgerald P, Dinan TG, Cryan JF. The probiotic Bifidobacterium infantis 35624 displays visceral antinociceptive effects in the rat. Neurogastroenterol Motil. 2010;22(9): 1029–35, e268. Epub 2010/06/04. doi: 10.1111/j.1365-2982.2010.01520.x . 20518856 10.1111/j.1365-2982.2010.01520.x

[62] Moya-Perez A, Perez-Villalba A, Benitez-Paez A, Campillo I, Sanz Y. Bifidobacterium CECT 7765 modulates early stress-induced immune, neuroendocrine and behavioral alterations in mice. Brain Behav Immun. 2017;65: 43–56. Epub 2017/05/18. doi: 10.1016/j.bbi.2017.05.011 . 28512033 10.1016/j.bbi.2017.05.011

[63] Smith CJ, Emge JR, Berzins K, Lung L, Khamishon R, Shah P, et al Probiotics normalize the gut-brain-microbiota axis in immunodeficient mice. Am J Physiol Gastrointest Liver Physiol. 2014;307(8): G793–802. Epub 2014/09/06. doi: 10.1152/ajpgi.00238.2014 ; PubMed Central PMCID: PMCPMC4200314. 25190473 10.1152/ajpgi.00238.2014 PMC4200314

[64] Vanhaecke T, Aubert P, Grohard PA, Durand T, Hulin P, Paul-Gilloteaux P, et al L. fermentum CECT 5716 prevents stress-induced intestinal barrier dysfunction in newborn rats. Neurogastroenterol Motil. 2017 Epub 2017/04/04. doi: 10.1111/nmo.13069 . 28370715 10.1111/nmo.13069

[65] Wang T, Hu X, Liang S, Li W, Wu X, Wang L, et al Lactobacillus fermentum NS9 restores the antibiotic induced physiological and psychological abnormalities in rats. Benef Microbes. 2015;6(5): 707–17. Epub 2015/04/15. doi: 10.3920/BM2014.0177 . 25869281 10.3920/BM2014.0177

[66] Sterne JA, Sutton AJ, Ioannidis JP, Terrin N, Jones DR, Lau J, et al Recommendations for examining and interpreting funnel plot asymmetry in meta-analyses of randomised controlled trials. BMJ. 2011;343: d4002 Epub 2011/07/26. doi: 10.1136/bmj.d4002 . 21784880 10.1136/bmj.d4002

[67] Moja L, Pecoraro V, Ciccolallo L, Dall'Olmo L, Virgili G, Garattini S. Flaws in animal studies exploring statins and impact on meta-analysis. Eur J Clin Invest. 2014;44(6): 597–612. Epub 2014/03/29. doi: 10.1111/eci.12264 . 24665945 10.1111/eci.12264

[68] Kato-Kataoka A, Nishida K, Takada M, Kawai M, Kikuchi-Hayakawa H, Suda K, et al Fermented Milk Containing Lactobacillus casei Strain Shirota Preserves the Diversity of the Gut Microbiota and Relieves Abdominal Dysfunction in Healthy Medical Students Exposed to Academic Stress. Appl Environ Microbiol. 2016;82(12): 3649–58. doi: 10.1128/AEM.04134-15 PubMed PMID: WOS:000377018400023. 27208120 10.1128/AEM.04134-15 PMC4959178

[69] Lorenzo-Zuniga V, Llop E, Suarez C, Alvarez B, Abreu L, Espadaler J, et al I.31, a new combination of probiotics, improves irritable bowel syndrome-related quality of life. World J Gastroenterol. 2014;20(26): 8709–16. Epub 2014/07/16. doi: 10.3748/wjg.v20.i26.8709 ; PubMed Central PMCID: PMCPMC4093724. 25024629 10.3748/wjg.v20.i26.8709 PMC4093724

[70] Lyra A, Hillila M, Huttunen T, Mannikko S, Taalikka M, Tennila J, et al Irritable bowel syndrome symptom severity improves equally with probiotic and placebo. World J Gastroenterol. 2016;22(48): 10631–42. Epub 2017/01/14. doi: 10.3748/wjg.v22.i48.10631 ; PubMed Central PMCID: PMCPMC5192275. 28082816 10.3748/wjg.v22.i48.10631 PMC5192275

[71] Marcos A, Warnberg J, Nova E, Gomez S, Alvarez A, Alvarez R, et al The effect of milk fermented by yogurt cultures plus Lactobacillus casei DN-114001 on the immune response of subjects under academic examination stress. Eur J Nutr. 2004;43(6): 381–9. Epub 2004/08/17. doi: 10.1007/s00394-004-0517-8 . 15309418 10.1007/s00394-004-0517-8

[72] Pinto-Sanchez MI, Hall GB, Ghajar K, Nardelli A, Bolino C, Lau JT, et al Probiotic Bifidobacterium longum NCC3001 Reduces Depression Scores and Alters Brain Activity: A Pilot Study in Patients With Irritable Bowel Syndrome. Gastroenterology. 2017;153(2): 448–59.e8. Epub 2017/05/10. doi: 10.1053/j.gastro.2017.05.003 . 28483500 10.1053/j.gastro.2017.05.003

[73] Romijn AR, Rucklidge JJ, Kuijer RG, Frampton C. A double-blind, randomized, placebo-controlled trial of Lactobacillus helveticus and Bifidobacterium longum for the symptoms of depression. Aust N Z J Psychiatry. 2017: 4867416686694 Epub 2017/01/11. doi: 10.1177/0004867416686694 . 28068788 10.1177/0004867416686694 PMC5518919

[74] Simren M, Ohman L, Olsson J, Svensson U, Ohlson K, Posserud I, et al Clinical trial: the effects of a fermented milk containing three probiotic bacteria in patients with irritable bowel syndrome—a randomized, double-blind, controlled study. Aliment Pharmacol Ther. 2010;31(2): 218–27. doi: 10.1111/j.1365-2036.2009.04183.x PubMed PMID: WOS:000272864600005. 19863495 10.1111/j.1365-2036.2009.04183.x

[75] Slykerman RF, Hood F, Wickens K, Thompson JMD, Barthow C, Murphy R, et al Effect of Lactobacillus rhamnosus HN001 in Pregnancy on Postpartum Symptoms of Depression and Anxiety: A Randomised Double-blind Placebo-controlled Trial. EBioMedicine. 2017;24: 159–65. Epub 2017/09/26. doi: 10.1016/j.ebiom.2017.09.013 ; PubMed Central PMCID: PMCPMC5652021. 28943228 10.1016/j.ebiom.2017.09.013 PMC5652021

[76] Steenbergen L, Sellaro R, van Hemert S, Bosch JA, Colzato LS. A randomized controlled trial to test the effect of multispecies probiotics on cognitive reactivity to sad mood. Brain Behav Immun. 2015 Epub 2015/04/12. doi: 10.1016/j.bbi.2015.04.003 . 25862297 10.1016/j.bbi.2015.04.003

[77] Takada M, Nishida K, Gondo Y, Kikuchi-Hayakawa H, Ishikawa H, Suda K, et al Beneficial effects of Lactobacillus casei strain Shirota on academic stress-induced sleep disturbance in healthy adults: a double-blind, randomised, placebo-controlled trial. Benef Microbes. 2017;8(2): 153–62. Epub 2017/04/27. doi: 10.3920/BM2016.0150 . 28443383 10.3920/BM2016.0150

[78] Takada M, Nishida K, Kataoka-Kato A, Gondo Y, Ishikawa H, Suda K, et al Probiotic Lactobacillus casei strain Shirota relieves stress-associated symptoms by modulating the gut-brain interaction in human and animal models. Neurogastroenterology and Motility. 2016;28(7): 1027–36. doi: 10.1111/nmo.12804 PubMed PMID: WOS:000383290800008. 26896291 10.1111/nmo.12804

[79] Yang H, Zhao X, Tang S, Huang H, Zhao X, Ning Z, et al Probiotics reduce psychological stress in patients before laryngeal cancer surgery. Asia Pac J Clin Oncol. 2016;12(1): e92–6. Epub 2014/02/28. doi: 10.1111/ajco.12120 . 24571169 10.1111/ajco.12120

[80] Harmer CJ, Cowen PJ. 'It's the way that you look at it'—a cognitive neuropsychological account of SSRI action in depression. Philos Trans R Soc Lond B Biol Sci. 2013;368(1615): 20120407 Epub 2013/02/27. doi: 10.1098/rstb.2012.0407 ; PubMed Central PMCID: PMCPMC3638386. 23440467 10.1098/rstb.2012.0407 PMC3638386

[81] Segers ME, Lebeer S. Towards a better understanding of Lactobacillus rhamnosus GG—host interactions. Microb Cell Fact. 2014;13 Suppl 1: S7 Epub 2014/09/05. doi: 10.1186/1475-2859-13-s1-s7 ; PubMed Central PMCID: PMCPMC4155824. 25186587 10.1186/1475-2859-13-S1-S7 PMC4155824

[82] Didari T, Solki S, Mozaffari S, Nikfar S, Abdollahi M. A systematic review of the safety of probiotics. Expert Opin Drug Saf. 2014;13(2): 227–39. Epub 2014/01/11. doi: 10.1517/14740338.2014.872627 . 24405164 10.1517/14740338.2014.872627

[83] Ibnou-Zekri N, Blum S, Schiffrin EJ, von der Weid T. Divergent patterns of colonization and immune response elicited from two intestinal Lactobacillus strains that display similar properties in vitro. Infect Immun. 2003;71(1): 428–36. Epub 2002/12/24. doi: 10.1128/IAI.71.1.428-436.2003 ; PubMed Central PMCID: PMCPMC143181. 12496193 10.1128/IAI.71.1.428-436.2003 PMC143181

[84] Wells JM. Immunomodulatory mechanisms of lactobacilli. Microb Cell Fact. 2011;10 Suppl 1: S17 Epub 2011/10/26. doi: 10.1186/1475-2859-10-s1-s17 ; PubMed Central PMCID: PMCPMC3231924. 21995674 10.1186/1475-2859-10-S1-S17 PMC3231924

[85] Lamouse-Smith ES, Tzeng A, Starnbach MN. The intestinal flora is required to support antibody responses to systemic immunization in infant and germ free mice. PLoS One. 2011;6(11): e27662 Epub 2011/11/25. doi: 10.1371/journal.pone.0027662 ; PubMed Central PMCID: PMCPMC3219679. 22114681 10.1371/journal.pone.0027662 PMC3219679

[86] Shapiro JM, Cho JH, Sands BE, LeLeiko NS. Bridging the gap between host immune response and intestinal dysbiosis in inflammatory bowel disease: does immunoglobulin A mark the spot? Clin Gastroenterol Hepatol. 2015;13(5): 842–6. Epub 2015/03/01. doi: 10.1016/j.cgh.2015.02.028 . 25725444 10.1016/j.cgh.2015.02.028

[87] Forsythe P, Kunze WA, Bienenstock J. On communication between gut microbes and the brain. Curr Opin Gastroenterol. 2012;28(6): 557–62. Epub 2012/09/27. doi: 10.1097/MOG.0b013e3283572ffa . 23010679 10.1097/MOG.0b013e3283572ffa

[88] Bebarta V, Luyten D, Heard K. Emergency medicine animal research: Does use of randomization and blinding affect the results? Acad Emerg Med. 2003;10(6): 684–7. Epub 2003/06/05. . 12782533 10.1111/j.1553-2712.2003.tb00056.x

[89] Crossley NA, Sena E, Goehler J, Horn J, van der Worp B, Bath PM, et al Empirical evidence of bias in the design of experimental stroke studies: A metaepidemiologic approach. Stroke. 2008;39(3): 929–34. Epub 2008/02/02. doi: 10.1161/STROKEAHA.107.498725 . 18239164 10.1161/STROKEAHA.107.498725

[90] Hirst JA, Howick J, Aronson JK, Roberts N, Perera R, Koshiaris C, et al The need for randomization in animal trials: an overview of systematic reviews. PLoS One. 2014;9(6): e98856 Epub 2014/06/07. doi: 10.1371/journal.pone.0098856 ; PubMed Central PMCID: PMCPMC4048216. 24906117 10.1371/journal.pone.0098856 PMC4048216

[91] Macleod MR, Lawson McLean A, Kyriakopoulou A, Serghiou S, de Wilde A, Sherratt N, et al Risk of Bias in Reports of In Vivo Research: A Focus for Improvement. PLoS Biol. 2015;13(10): e1002273 Epub 2015/10/16. doi: 10.1371/journal.pbio.1002273 ; PubMed Central PMCID: PMCPMC4603955. 26460723 10.1371/journal.pbio.1002273 PMC4603955

[92] Lozupone CA, Stombaugh JI, Gordon JI, Jansson JK, Knight R. Diversity, stability and resilience of the human gut microbiota. Nature. 2012;489(7415): 220–30. Epub 2012/09/14. doi: 10.1038/nature11550 ; PubMed Central PMCID: PMCPMC3577372. 22972295 10.1038/nature11550 PMC3577372

[93] Bogovic Matijasic B, Obermajer T, Lipoglavsek L, Sernel T, Locatelli I, Kos M, et al Effects of synbiotic fermented milk containing Lactobacillus acidophilus La-5 and Bifidobacterium animalis ssp. lactis BB-12 on the fecal microbiota of adults with irritable bowel syndrome: A randomized double-blind, placebo-controlled trial. J Dairy Sci. 2016;99(7): 5008–21. Epub 2016/05/10. doi: 10.3168/jds.2015-10743 . 27157575 10.3168/jds.2015-10743

[94] McNulty NP, Yatsunenko T, Hsiao A, Faith JJ, Muegge BD, Goodman AL, et al The impact of a consortium of fermented milk strains on the gut microbiome of gnotobiotic mice and monozygotic twins. Sci Transl Med. 2011;3(106): 106ra Epub 2011/10/28. doi: 10.1126/scitranslmed.3002701 ; PubMed Central PMCID: PMCPMC3303609. 22030749 10.1126/scitranslmed.3002701 PMC3303609

[95] Koren O, Goodrich JK, Cullender TC, Spor A, Laitinen K, Backhed HK, et al Host remodeling of the gut microbiome and metabolic changes during pregnancy. Cell. 2012;150(3): 470–80. Epub 2012/08/07. doi: 10.1016/j.cell.2012.07.008 ; PubMed Central PMCID: PMCPMC3505857. 22863002 10.1016/j.cell.2012.07.008 PMC3505857

[96] DiGiulio DB, Callahan BJ, McMurdie PJ, Costello EK, Lyell DJ, Robaczewska A, et al Temporal and spatial variation of the human microbiota during pregnancy. Proc Natl Acad Sci U S A. 2015;112(35): 11060–5. Epub 2015/08/19. doi: 10.1073/pnas.1502875112 ; PubMed Central PMCID: PMCPMC4568272. 26283357 10.1073/pnas.1502875112 PMC4568272

[97] Labus JS, Bolus R, Chang L, Wiklund I, Naesdal J, Mayer EA, et al The Visceral Sensitivity Index: development and validation of a gastrointestinal symptom-specific anxiety scale. Aliment Pharmacol Ther. 2004;20(1): 89–97. Epub 2004/07/01. doi: 10.1111/j.1365-2036.2004.02007.x . 15225175 10.1111/j.1365-2036.2004.02007.x

[98] Jerndal P, Ringstrom G, Agerforz P, Karpefors M, Akkermans LM, Bayati A, et al Gastrointestinal-specific anxiety: an important factor for severity of GI symptoms and quality of life in IBS. Neurogastroenterol Motil. 2010;22(6): 646–e179. Epub 2010/04/07. doi: 10.1111/j.1365-2982.2010.01493.x . 20367800 10.1111/j.1365-2982.2010.01493.x

[99] Melsen WG, Bootsma MC, Rovers MM, Bonten MJ. The effects of clinical and statistical heterogeneity on the predictive values of results from meta-analyses. Clin Microbiol Infect. 2014;20(2): 123–9. Epub 2013/12/11. doi: 10.1111/1469-0691.12494 . 24320992 10.1111/1469-0691.12494

[100] Button KS, Ioannidis JP, Mokrysz C, Nosek BA, Flint J, Robinson ES, et al Power failure: why small sample size undermines the reliability of neuroscience. Nat Rev Neurosci. 2013;14(5): 365–76. Epub 2013/04/11. doi: 10.1038/nrn3475 . 23571845 10.1038/nrn3475

