# Supplementary material for: The anxiolytic effect of probiotics: A systematic review and meta-analysis of the clinical and preclinical literature
Source: PLoS One. 2018 Jun 20;13(6):e0199041. doi: 10.1371/journal.pone.0199041 (PMC6010276; doi:10.1371/journal.pone.0199041)
Supplement: S1 Appendix — (DOCX) [file pone.0199041.s001.docx]

**S1 Appendix.** Preclinical and Clinical PubMed Search Algorithm

| Step | Preclinical Search Terms |
| --- | --- |
| 1 | Rat* or mouse* or mice* or murine or rodent |
| 2 | Bifidobacterium or Probiotic* or Lactobacillus or prebiotic* or synbiotic* or saccharomyces or mycobacterium |
| 3 | 1 and 2 |
| 4 | Anxiety or anxious or anx* or anxiety-like |
| 5 | mental health* |
| 6 | Psycholo* and stress |
| 7 | 4 or 5 or 6 |
| 8 | 3 and 7 |

| Step | Clinical Search Terms |
| --- | --- |
| 1 | randomized controlled trial* or Clinical Trial * or trial |
| 2 | Bifidobacterium or Bacteria* or Probiotic* or Lactobacillus or prebiotic* or synbiotic* or saccharomyces |
| 3 | 1 and 2 |
| 4 | Anxiety or anxious or anxio* |
| 5 | mental health* |
| 6 | Psycholo* and stress |
| 7 | 4 or 5 or 6 |
| 8 | 3 and 7 |
