# Supplementary material for: The anxiolytic effect of probiotics: A systematic review and meta-analysis of the clinical and preclinical literature
Source: PLoS One. 2018 Jun 20;13(6):e0199041. doi: 10.1371/journal.pone.0199041 (PMC6010276; doi:10.1371/journal.pone.0199041)
Supplement: S2 Appendix — (DOCX) [file pone.0199041.s002.docx]

Variable Dictionary

| Variable | Description |
| --- | --- |
| study | Study number |
| author | Author name |
| year | Year of publication |
| tx_dur | Duration of probiotic treatment in days |
| tx_cfu_billions | Probiotic treatment dose in billions of colony-forming units |
| tx_n | Sample size of treatment group |
| c_n | Sample size of control group |
| hedges | Standardized mean difference between treatment and control group (Hedge’s g) – Negative value = greater anxiety reduction in treatment group |
| se | Standard error of standardized mean difference |
|  |  |
| Preclinical specific variables | |
| paradigm | Behavioral paradigm |
| measure | Specific paradigm measure to assess for anxiety-like behavior |
| rodent | Rodent species employed – mouse or rat |
| clinical | Animal model of disease (0 = no, 1 = yes) |
|  |  |
| Clinical specific variables | |
| scale | Anxiety scale |
| clinical | Clinical sample (0 = no, 1 = yes) |
