# Supplementary material for: The anxiolytic effect of probiotics: A systematic review and meta-analysis of the clinical and preclinical literature
Source: PLoS One. 2018 Jun 20;13(6):e0199041. doi: 10.1371/journal.pone.0199041 (PMC6010276; doi:10.1371/journal.pone.0199041)

Studies	SMD (95% CI)	Weight (%)
**Agusti, A. 2017 Group 1**		
OFT center time	−0.49 (−1.38, 0.40)	1.6
LDT latency to enter light zone	0.56 (−0.34, 1.46)	1.6
**Agusti, A. 2017 Group 2**		
OFT center time	−0.59 (−1.49, 0.31)	1.6
LDT latency to enter light zone	−0.43 (−1.32, 0.46)	1.6
**Barrera−Bugueno, C. 2017**		
OFT center time	5.39 (3.19, 7.59)	0.4
OFT center entries	6.66 (4.03, 9.29)	0.4
EPM open arm time	2.83 (1.43, 4.23)	0.4
EPM open arm entries	1.13 (0.11, 2.15)	0.4
**Beilharz, J. 2017 Group 1**		
EPM open arm time	0.74 (−0.05, 1.53)	3.5
**Beilharz, J. 2017 Group 2**		
EPM open arm time	−0.01 (−0.78, 0.76)	3.5
**Bercik, P. 2010**		
LDT time in light zone	−0.57 (−1.21, 0.07)	1.2
LDT latency to re−enter light zone	−0.39 (−1.02, 0.24)	1.2
Step−down latency	−1.01 (−1.67, −0.35)	1.2
**Bercik, P. 2011 Group 1**		
Step−down latency	−1.56 (−2.52, −0.60)	3.1
**Bercik, P. 2011 Group 2**		
Step−down latency	0.31 (−0.41, 1.03)	3.6
**Bharwani, A. 2017 Group 1**		
LDT light zone entries	0.00 (−0.73, 0.73)	3.6
**Bharwani, A. 2017 Group 2**		
LDT light zone entries	−1.22 (−2.00, −0.44)	3.5
**Bravo, J. 2011**		
EPM open arm entries	−1.49 (−2.24, −0.74)	1.8
EPM open arm time	−0.43 (−1.10, 0.24)	1.8
**Cowan, C. 2016**		
EPM open arm entries	0.30 (−0.69, 1.29)	1.0
EPM open arm time	−0.28 (−1.27, 0.71)	1.0
EPM latency to enter open arms	0.02 (−0.96, 1.00)	1.0
**Divyashri, G. 2015**		
EPM open arm entries	−2.22 (−4.41, −0.03)	0.3
EPM open arm time	−1.15 (−3.67, 1.37)	0.3
OFT center entries	0.65 (−0.58, 1.88)	0.3
OFT center time	−1.39 (−3.18, 0.40)	0.3
**Emge, J. 2016 Group 1**		
LDT time in light zone	0.99 (−0.39, 2.37)	2.3
**Emge, J. 2016 Group 2**		
LDT time in light zone	−1.07 (−2.02, −0.12)	3.1
**Jang, H. 2017**		
EPM open arm time	−1.81 (−2.89, −0.73)	1.4
EPM open arm entries	−2.20 (−3.35, −1.05)	1.4
**Liang S. 2015**		
EPM open arm time	−1.39 (−2.52, −0.26)	0.9
EPM open arm entries	−1.49 (−2.64, −0.34)	0.9
OFT center time	−1.54 (−2.70, −0.38)	0.9
**Liu, W. 2016 Group 1**		
EPM time (open/closed)	−1.22 (−2.19, −0.25)	1.5
OFT center time	0.55 (−0.35, 1.45)	1.5
**Liu, W. 2016 Group 2**		
OFT center time	−0.77 (−1.96, 0.42)	1.2
EPM open arm time	−1.54 (−2.90, −0.18)	1.2
**Liu, Y. 2016 Group 1**		
OFT center time	0.48 (−0.41, 1.37)	1.6
EPM open arm time	0.59 (−0.31, 1.49)	1.6
**Liu, Y. 2016 Group 2**		
OFT center time	−1.17 (−2.19, −0.15)	1.4
EPM open arm time	−1.63 (−2.73, −0.53)	1.4
**Luo, J. 2014**		
EPM open time	−3.13 (−5.03, −1.23)	0.8
EPM open arm entries	−3.22 (−5.16, −1.28)	0.8
**Mackos, A. 2013 Group 1**		
OFT center time	−0.01 (−0.66, 0.64)	3.7
**Mackos, A. 2013 Group 2**		
OFT center time	−0.42 (−1.36, 0.52)	3.1
**Matthews, D. 2013**		
Immobilization	−0.90 (−1.89, 0.09)	1.0
Grooming	−0.67 (−1.63, 0.29)	1.0
Latency to start	−0.51 (−1.46, 0.44)	1.0
**McKernan, D. 2010 Group 1**		
OFT center time	−0.47 (−1.19, 0.25)	3.6
**McKernan, D. 2010 Group 2**		
OFT center time	−0.11 (−0.83, 0.61)	3.6
**Moya−Perez, A. 2017 Group 1**		
EPM open arm time	0.36 (−0.57, 1.29)	3.2
**Moya−Perez, A. 2017 Group 2**		
EPM open arm time	−1.12 (−2.26, 0.02)	1.4
OFT center entries	−0.20 (−1.13, 0.73)	1.4
**Smith, C. 2014 Group 1**		
LDT time in light zone	−1.23 (−2.57, 0.11)	2.4
**Smith, C. 2014 Group 2**		
LDT time in light zone	−1.97 (−3.63, −0.31)	1.9
**Vanhaecke, T. 2017**		
EPM open arm entries	−0.34 (−1.48, 0.80)	2.7
**Wang, T. 2015**		
EPM open arm entries	−0.59 (−1.49, 0.31)	3.2
**Summary** (I^2 = 70.54%)	**−0.47 (−0.77, −0.16)**	

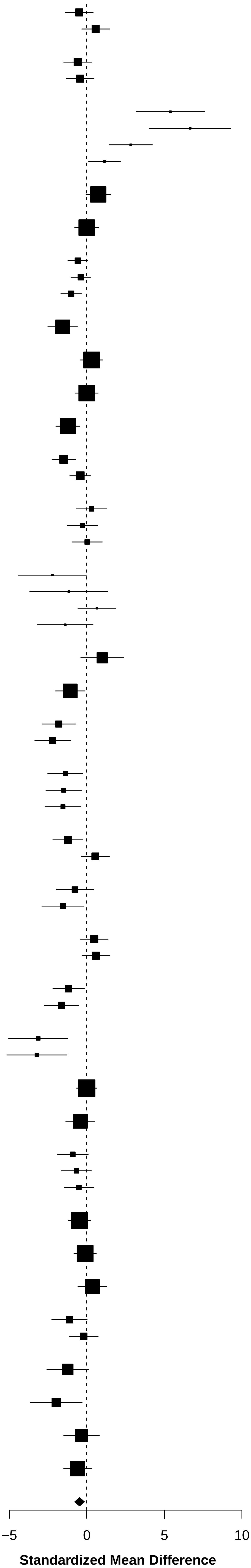

Supplement: S1 Fig — SMD = Standardized mean difference; CI = Confidence interval; EPM = Elevated plus maze; LDT = Light-dark test; Step-down = Step-down test; OFT = Open field test. (PDF) [file pone.0199041.s003.pdf]
